# The telomere‐to‐telomere gap‐free reference genome and DNA methylome unveil epigenetic regulation of proanthocyanidin biosynthesis during apricot fruit development

**DOI:** 10.1111/tpj.71138

**Published:** 2026-09-22

**Authors:** Xu Qiang, Tingting Zhang, Meng Wang, Ting Ren, Guoqing Bai, Ying Zhang, Zhikun Chen, Xiying Du, Yun Jia, Zhonghu Li, Ming Yue

**Affiliations:** ^1^ Xi'an Botanical Garden of Shaanxi Province (Institute of Botany of Shaanxi Province) Xi'an Shaanxi 710061 China; ^2^ Key Laboratory of Resource Biology and Biotechnology in Western China (Ministry of Education) College of Life Sciences Northwest University Xi'an Shaanxi 710069 China; ^3^ College of Life Science and Technology Gansu Agricultural University Lanzhou Gansu 730070 China; ^4^ College of Environment and Life Sciences Weinan Normal University Weinan Shaanxi 714099 China; ^5^ Xi'an Institute of Plums and Apricots Xi'an Shaanxi 710308 China

**Keywords:** *Prunus armeniaca*, T2T genome, multi‐omics, DNA methylome, metabolic regulatory network, transcription factor

## Abstract

Apricot (*Prunus armeniaca* L.) is an economically significant fruit crop worldwide with remarkable germplasm diversity, and its fruit quality is primarily determined by key metabolites (e.g., sugars, organic acids, and flavonoids). However, current apricot genome assemblies remain incomplete, with substantial unanchored regions that hamper analyses of genomic evolution and the regulatory mechanisms underlying fruit quality. Here, we generated the first gap‐free telomere‐to‐telomere (T2T) *de novo* assembly of the *P. armeniaca* genome (248.3 Mb) and annotated 22 786 protein‐coding genes across eight chromosomes. Combined analyses of metabolomic and transcriptomic data, we characterized the transcriptomic and metabolic landscape of apricot across five developmental stages and assigned 3198 metabolites and their co‐expressed genes to five functional metabolic‐gene modules. DNA methylome profiling further revealed dynamic epigenetic reprogramming during fruit development, characterized by decreases in CG and CHG methylation and an increase in CHH methylation. Notably, higher CHH DNA methylation in promoter regions was associated with the upregulation of leucoanthocyanidin reductase (*PaLAR*) and dihydroflavonol 4‐reductase (*PaDFR*), contributing to anthocyanin biosynthesis during ripening. Moreover, we identified *PaTT2* as a potential regulator of proanthocyanidin biosynthesis and directly activates *PaLAR*, as evidenced by Y1H and dual‐luciferase assays. Our study sheds light on the epigenetic and metabolic regulatory networks of apricot, serving as a valuable foundation for molecular breeding and genetic enhancement.

## Significance Statement

Apricot is an economically important fruit crop valued for its flavor and nutritional benefits. We present the first gap‐free T2T apricot genome, providing a valuable resource for genomic studies. Integrated transcriptomic, metabolomic, and DNA methylation analyses revealed dynamic changes during fruit ripening and identified potential regulatory modules, such as PaTT2–PaLAR, associated with proanthocyanidin biosynthesis. These findings suggest a potential regulatory role of DNA methylation in fruit quality formation.

## INTRODUCTION

Apricot (*Prunus armeniaca* L.) and the cultivated species (also called “common apricot”), belonging to the genus *Prunus* in the Rosaceae family, are one of the most economically significant stone fruit crops worldwide (Groppi et al., 2021). Its fruits are widely consumed fresh and processed into various products such as canned goods and beverages, with an annual global production of 4.1 million tons (Groppi et al., 2021). Accordingly, fruit quality is a critical breeding objective that highly affects consumer appreciation and market competitiveness. Apricot fruits are enriched in health‐promoting metabolites with high antioxidant activity and unique aroma, exhibiting a characteristic orange color primarily attributed to the accumulation of carotenoids, particularly β‐carotene (Jiang et al., 2019; Sass‐Kiss et al., 2005). Aside from its excellent flavor and nutritional attributes, apricot also possesses high ornamental and cultural value, reflecting over 3000 years of cultivation in China (Jiang et al., 2019). Its long‐standing domestication has generated extensive germplasm diversity, providing a valuable reservoir for genetic improvement and crop adaptation (Zhang et al., 2021). However, in most commercially grown cultivars of *P. armeniaca*, the genetic mechanisms underlying the evolutionary dynamics and fruit quality traits remain poorly understood.

The fruits of many members of the *Prunus* are edible, and the genus includes economically significant crops such as *P. armeniaca* (“Xiaobaixing,” “Yinxiangbai”), and the wild species *P. zhengheensis* from warm Southeast China, for which high‐quality genome assemblies have recently been reported (Guo et al., 2023; Jiang et al., 2019; Tan et al., 2024; Zhang et al., 2021). Genome sequencing serves as a robust approach to uncover the genetic basis of evolutionary history and functional genomics studies. Nevertheless, these earlier apricot reference genomes still contain numerous unclosed gaps and unanchored sequences, particularly within highly repetitive regions. For instance, in the latest upgraded reference genome of *P. armeniaca* that has been released (namely, Prunus_zhengheensis_hap1), 87 unresolved gaps and the telomeres and centromeres are not mentioned at all (Tan et al., 2024). To date, advances in long‐read sequencing technologies and assembling tools have enabled the construction of telomere‐to‐telomere (T2T) genome assemblies in many model plants and horticultural crops, such as Arabidopsis (Naish et al., 2021), rice (Song et al., 2021), chili pepper (Chen et al., 2024), and linseed (Yadav et al., 2025). T2T assembly refers to the generation of complete genome sequences that decipher all centromeres and highly repetitive regions (Nurk et al., 2022). The incompleteness of the apricot genome has hindered studies of its genetic and molecular mechanisms, and a high‐quality, gap‐free genome assembly is indispensable.

The key determinants of fruit quality are primarily associated with traits such as aroma, color, taste (sweetness and acidity), and nutritional composition (Zeng et al., 2025). Among these, soluble sugars (e.g., sucrose, glucose, and fructose) and organic acids are primary contributors to palatability, while bioactive compounds such as flavonoids with antioxidant activity play a critical role in supporting human health (Zhong et al., 2024). Given the significance of metabolites to apricot quality, multi‐omics approaches (e.g., metabolomic and transcriptomic profiling) have recently proven highly effective in identifying regulatory genes in flavor‐ and nutrition‐related pathways. For instance, variations in aroma, soluble sugars, and organic acids have been observed among different apricot cultivars (e.g., “Shushanggan,” “Wuyuexian” and “Luntaibaixing”) (Gou et al., 2023; He et al., 2025; Zeng et al., 2025). Several structural genes and transcription factors (TFs), such as bHLH and MYB, have been shown to contribute to flavonoid accumulation (Chen et al., 2023; Xi et al., 2019). Fruit formation involves a set of highly complicated, well‐coordinated and finely metabolic dynamic process generally determined by the key metabolites and underlying regulatory networks (He et al., 2025). Hence, a systematic investigation of the genetic and epigenetic mechanisms underlying metabolite biosynthesis during fruit development is essential for improving apricot breeding and fruit quality.

DNA methylation is a pivotal epigenetic marker that regulates multiple biological processes in plants, including seed germination, fruit development and ripening, and responses to environmental stresses (Law & Jacobsen, 2010; Zhang et al., 2018). Emerging evidence indicates that dynamic DNA methylation changes are critical for fruit development (Gapper et al., 2013; Huang et al., 2019). For example, in sweet orange, a global increase in DNA methylation during fruit ripening significantly affects physiological processes such as photosynthesis (Huang et al., 2019). In blood orange, cold‐induced alterations in DNA methylation patterns modulate the expression of anthocyanin biosynthesis genes (Sicilia et al., 2020). Similarly, in lemon, elevated CHH methylation in the *ClPEPCK* promoter correlates with its high expression, suggesting a pivotal player in regulating citric acid synthesis (Yu et al., 2024). Collectively, these findings highlight the central role of DNA methylation in regulating fruit metabolism and quality traits. However, genome‐wide DNA methylation dynamics and their functional roles in apricot remain largely unexplored.

In this study, we employed ultra‐long Oxford Nanopore Technology (ONT), PacBio sequencing, and Hi‐C to generate a gap‐free T2T genome of *P. armeniaca*, the newly developed cultivar “Fengyuanxing” (Fyx; “Chuanzhihong”♀ × “Jintaiyang”♂), adapted to Northwest China. Moreover, we applied a multi‐omics strategy to generate metabolomes, transcriptomes, and DNA methylation datasets across apricot fruit development. Integrative bioinformatics analyses and functional genomics assays allowed us to establish a metabolic regulatory network mapping the transcriptional and epigenetic regulation of major metabolite biosynthesis in apricot. This study advances insights into genome evolution and DNA methylation‐mediated fruit development, laying the foundation for genetic and epigenetic improvement of apricot cultivars.

## RESULTS

### T2T gap‐free reference genome for apricot

**Figure 1:**
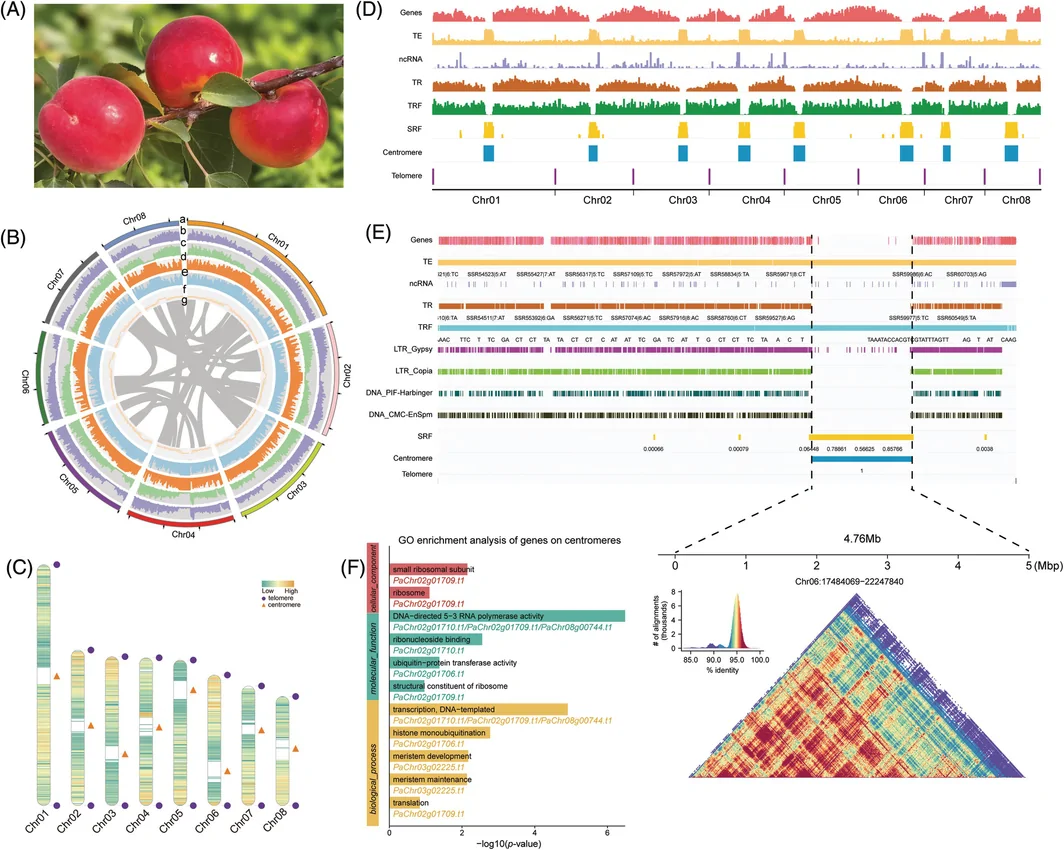
Phenotypic and genomic characterization of *Prunus armeniaca* Fyx. (A) Representative fruit morphology. (B) Circos plot visualizing genomic architecture. Tracks (outer to inner): (a) Pseudo‐chromosome ideograms; (b) Gene counts; (c) Gene density (genes per Mb); (d) Repetitive sequence density (% per Mb); (e) Transposable element (TE) density (% per Mb); (f) GC content (%); (g) Syntenic block relationships. (C) Visualization for the positions of telomeres and centromeres. (D) Distribution of genes, transposable elements (TEs), non‐coding RNAs (ncRNAs), tandem repeats (TRs), telomeric repeat factors (TRFs), satellite repeat factors (SRFs), centromeres, and telomeres in the whole genome. (E) Visualization of the predicted centromeric region on chromosome 6. The triangular heatmap depicts intra‐haplotype sequence identity, color‐coded by percentage similarity. (F) GO enrichment of the genes on centromeres.

*P. armeniaca* Fyx, a cultivated variety of apricot selected for its superior edibility, was used for T2T genome assembly (Figure 1A). On the whole, the assembly leveraged integrated sequencing data, including 14.8 Gb (∼60×) PacBio HiFi reads, 31.6 Gb (∼127.5×) ultra‐long ONT reads, 31.6 Gb (∼127.5×) Hi‐C chromatin conformation capture data, and 16.7 Gb (∼67.7×) Illumina short‐read data. All sequencing datasets achieved a minimum mapping rate exceeding 91.0% (Table S1). Genome survey analysis of *P*. *armeniaca* Fyx using k‐mer frequency distribution of short reads revealed an estimated genome size of 270.95 Mb, with the heterozygosity for 1.39% and the repetitive sequence content of 48.62% (Figure S1). The *de novo* assembly of the *P*. *armeniaca* Fyx genome achieved a contig N50 of 26.43 Mb. The scaffold N50 was 30.88 Mb with only two gaps remaining in the initial assembly (Table S2). Furthermore, the Hi‐C interaction heatmap demonstrated uniformly high intrachromosomal interaction intensities across all chromosomes, with no detectable aberrant *trans*‐chromosomal signals (Figure S2). The statistics of the eight pseudochromosomes identified two unresolved gaps, located on chromosome 2 (Chr02: 17750664–17750764) and chromosome 4 (Chr04: 11367227–11367327), respectively (Table S3). By sequence alignment, 264 and 337 PacBio HiFi reads, together with 251 and 440 ONT ultra‐long reads, matched to the gaps on chromosomes 2 and 4, respectively, with most reads spanning both flanking regions (Figure S3). Following successful gap filling using ONT ultra‐long and PacBio HiFi reads, a final gap‐free genome assembly of 248.31 Mb with a contig N50 of 30.63 Mb was achieved (Figure S4; Table S4). And after further polish, the coverage rates for all chromosomes exceeded 96% (Table S5). BUSCO assessment demonstrated that 99.69% of the core conserved plant orthologs were retained in the chromosome‐scale assembly (Table S6). Compared with 11 previously published apricot genomes assembly, our chromosome‐scale assembly exhibits comparable size while achieving T2T completeness with no gaps, absence of ambiguous bases, and unambiguous annotation of centromeres and telomeres on all chromosomes, along with the highest long‐terminal repeat (LTR) Assembly Index (LAI = 20.53) and BUSCO completeness score among all currently available assemblies (Table S7). RNA‐seq read alignment against the assembly showed a mapping rate of approximately 98.14%. Collectively, these results collectively confirm that the final apricot T2T and gap‐free genome assembly exhibits exceptional reliability and quality.

### Genome annotation and detection of telomeres and centromeres

For transposable elements (TEs), protein‐coding genes, TFs, and non‐coding RNAs of the *P. armeniaca* genome were annotated by integrating multiple approaches. Genome annotation showed that repetitive sequences account for 46.70% (115.95 Mb) of the assembled genome, with TEs representing the major fraction (43.82%). Among them, LTR elements, especially those of the Gypsy subfamily, constituted the most abundant class in the genome (Table S8). LTR retrotransposons were the most common type and comprised 18.49% of the genome. A total of 22 786 protein‐coding genes were annotated through harmonized multi‐database integration (Table S9). A total of 99.69% completely assembled genes and 97.1% complete proteins were assessed by the BUSCO analysis (Table S10). Specifically, 1364 TFs were identified among 68 families in the apricot, including 116 MYB, 105 NAC, 100 bHLH, 84 C2H2, 77 AP2/ERF, 62 C3H, 57 WRKY, 50 MYB‐related, 47 bZIP, and 47 GRAS members (Table S11). The prediction of non‐coding RNA (ncRNA) genes in the *P*. *armeniaca* Fyx genome identified 1509 ribosomal RNAs (rRNAs), 372 small nuclear RNAs (snRNAs), 86 microRNAs (miRNAs), 3 regulatory RNAs, and 534 transfer RNAs (tRNAs), which accounted for 1.50% of the genome (Table S12).

Telomeres are regions of highly repetitive DNA located at chromosome ends, which protect chromosomes from fraying or fusion (Shakirov et al., 2022). In plants, telomeric sequences are highly conserved, consisting of a 7‐bp repeat unit (CCCATTT at the 5′ end and TTTAGGG at the 3′ end) (Fajkus et al., 2005). We checked the 150‐kb sequences at both ends of each chromosome, and using the seven‐base telomeric repeat as a sequence query, we identified 16 telomeres on the terminals of the 8 chromosomes (Table S13). Additionally, the precise locations and lengths of centromeres across all chromosomes were quantified, with centromere sizes ranging from 2.3 to 4.7 Mb. Chromosome 6 exhibits the longest centromeric region among all chromosomes (Table S14). Concurrently, it was observed that the centromeric regions exhibited significantly lower gene number and gene density compared with other regions on chromosomes (Figure 1B,C). We also observed a marked enrichment of TEs and satellite repeat factors (SRFs) in centromeric regions compared with gene distribution patterns (Figure 1D,E; Figures S6–S8). To identify candidate genes within these genomic segments, we subsequently screened all annotated genes across the highly linked centromeric domains. Interestingly, we found 29 genes, with the majority annotated through the KOG and NR databases (Table S15). Functional annotation analysis based on gene ontology (GO) revealed the enrichment of two genes in ribosomal composition (cellular component). In addition, we found six genes enriched in molecular function catalog, such as DNA‐directed 5′–3′ RNA polymerase activity, ribonucleoside binding, ubiquitin–protein transferase activity, and structural constituent of ribosome. Moreover, transcription, DNA‐templated, histone monoubiquitination, meristem development, and translation appeared enriched in biological process‐related terms (Figure 1F; Table S16). Given the small set of genes analyzed, the enrichment results provide suggestive insights into potential functional categories.

### Comparative genomic analysis of *Prunus* genomes

**Figure 2:**
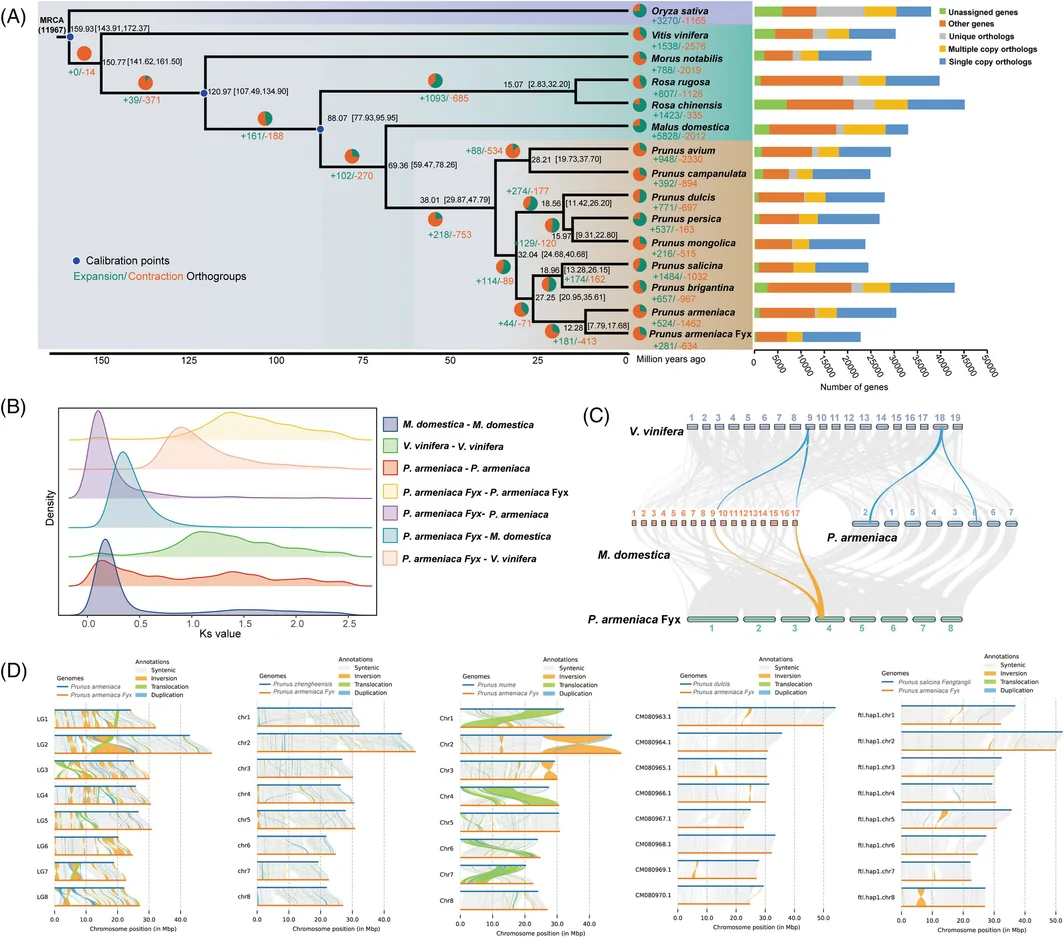
Comparative genome of *Prunus armeniaca* Fyx. (A) phylogenetic tree, gene family contraction/expansion, and genome clustering of orthologous across 15 species. (B) The synonymous substitution rate distribution among Fyx, *M*. *domestica*, *P*. *armeniaca*, and *V*. *vinifera*. (C) The syntenic relationship among *P*. *armeniaca* Fyx, *M*. *domestica*, *P*. *armeniaca*, and *V*. *vinifera*. (D) Structural variants between Fyx and the other five related *Prunus* genomes (*P*. *armeniaca*, *P*. *zhengheensis*, *P*. *mume*, *P*. *dulcis*, and *P*. *salicina* Fengtangli).

To elucidate genome‐wide evolution within apricots, we conducted comparative genomic analyses with 15 species, including model species and published relatives. Genome clustering of orthologous gene families across 15 species identified 6454 conserved clusters, including 569 universal single‐copy orthogroups shared by all involved species. The results revealed conserved orthogroup distribution between Fyx and *P*. *armeniaca*, with both species exhibiting comparable quantities of single‐copy and multi‐copy orthologs. Notably, the number of other genes in *P*. *armeniaca* was significantly greater than that in Fyx. In contrast, these values were significantly distinct from those observed in *M*. *domestica*, indicating lineage‐specific gene duplication dynamics within Rosaceae (Figure 2A).

Phylogenetic analysis revealed that within the Rosaceae family, *M*. *domestica* diverged approximately 69.36 million years ago (Mya), while the *Prunus* genus diverged around 38.01 Mya. Within *Prunus sensu lato*, three major evolutionary clades were identified, with Fyx and *P*. *armeniaca* exhibiting a closer evolutionary relationship. And the divergence time between the two apricots was estimated to be 12.28 Mya (Figure 2A). To understand gene family evolutionary dynamics, we conducted comparative genomic analysis using CAFE. A total of 11 967 gene families were inferred at the most recent common ancestor (MRCA) of *Prunus* species, from which 281 expanded and 634 contracted families were identified in the *P. armeniaca* Fyx genome (Figure 2A). GO enrichment analysis indicated that expanded gene families were significantly enriched in the following categories: defense response, ubiquitin‐dependent protein catabolic process, ubiquitin–protein transferase activator activity, and fatty‐acyl‐CoA reductase (alcohol‐forming) activity. Meanwhile, the results of KEGG enrichment further revealed significantly enriched categories in isoquinoline alkaloid biosynthesis, biosynthesis of secondary metabolites‐unclassified, and ubiquitin‐mediated proteolysis (Figure S9A; Tables S17 and S18). Conversely, the contracted gene families were predominantly associated with GO terms related to carbohydrate metabolism, oxidative stress response, and abscisic acid signaling. KEGG enrichment showed that these families were primarily involved in pyruvate metabolism and phenylpropanoid biosynthesis pathways (Figure S9B; Tables S19 and S20).

Ks‐based analysis of synonymous substitution rates supported an ancestral whole‐genome duplication (WGD) event prior to the *Vitis*–*Prunus* divergence, with paralogous gene retention signatures pervasive in *P*. *armeniaca* Fyx and *M*. *domestica* (Figure 2B). Integrating Ks distributions and syntenic alignments, we observed a lineage‐specific WGD in *M*. *domestica*, unequivocally distinct from the other three involved species (Figure 2C; Figure S9C). Duplication mode analysis further showed that WGD constituted the largest proportion of recently duplicated genes, accounting for 47% of all duplicated genes in *M. domestica*, followed by dispersed duplications (DSD, 19%), proximal duplications (PD, 5%), and tandem duplication (TD, 4%) (Figure S9C). Comparative analysis of Ks distributions revealed high concordance between Fyx and *P*. *armeniaca*, with the exception of a localized peak at Ks ≈ 0.2 in *P*. *armeniaca*. This species‐specific signature, suggestive of segmental gene duplications, is further substantiated by synteny analysis (Figure 2B,C).

Through integrated synteny‐based alignment and structural variant screening, we systematically characterized conserved syntenic blocks, segmental duplications (SDs), interchromosomal translocations, inversions, and unaligned genomic segments between *P*. *armeniaca* Fyx and other *Prunus* species. The results revealed extensive syntenic conservation between *P*. *armeniaca* Fyx and five *Prunus* species, with collinear blocks spanning 165.43 Mb (*P. zhengheensis*), 155.78 Mb (*P. dulcis*), 147.37 Mb (*P*. *salicina* Fengtangli), 124.98 Mb (*P*. *armeniaca*), and 107.18 Mb (*P. mume*), reflecting evolutionary conservation in specific chromosomal regions (Table S20). Furthermore, several genomic rearrangements such as inversions (45.39 Mb in *P*. *armeniaca*; 27.30 Mb in *P*. *mume*; 5.74 Mb in *P*. *salicina* Fengtangli; 3.03 Mb in *dulcis*; 2.87 Mb in *P*. *zhengheensis*), translocations (41.54 Mb in *P*. *mume*; 25.69 Mb in *P*. *armeniaca*; 10.60 Mb in *P*. *zhengheensis*; 2.79 Mb in *P*. *salicina* Fengtangli; 1.22 Mb in *dulcis*), and SDs (14.54 Mb in *P*. *armeniaca*; 14.05 Mb in *P*. *mume*; 13.77 Mb in *P*. *zhengheensis*; 7.59 Mb in *P*. *salicina* Fengtangli; 5.16 Mb in *dulcis*) were detected, collectively highlighting divergent evolutionary trajectories in genomic architecture. Our results also revealed substantial unaligned genomic segments across species comparisons (103.06 Mb in *P. dulcis*; 100.97 Mb in *P*. *salicina* Fengtangli; 51.16 Mb in *P*. *mume*; 34.05 Mb in *P*. *zhengheensis*; 6.95 Mb in *P*. *armeniaca*), which reflect lineage‐specific structural divergence and contribute to the genetic complexity within the *Prunus* genus (Figure 2D; Table S21). Annotation of these structural variation regions revealed that the sequences primarily consisted of SNPs, deletions, copylosses sequences, highly diverged sequences, and tandem repeats (TRs). In total, these results provide insights into genomic architecture and evolutionary relationships, highlighting the role of genomic rearrangements in species evolution and adaptation.

### Observation and characterization of apricot fruit development

**Figure 3:**
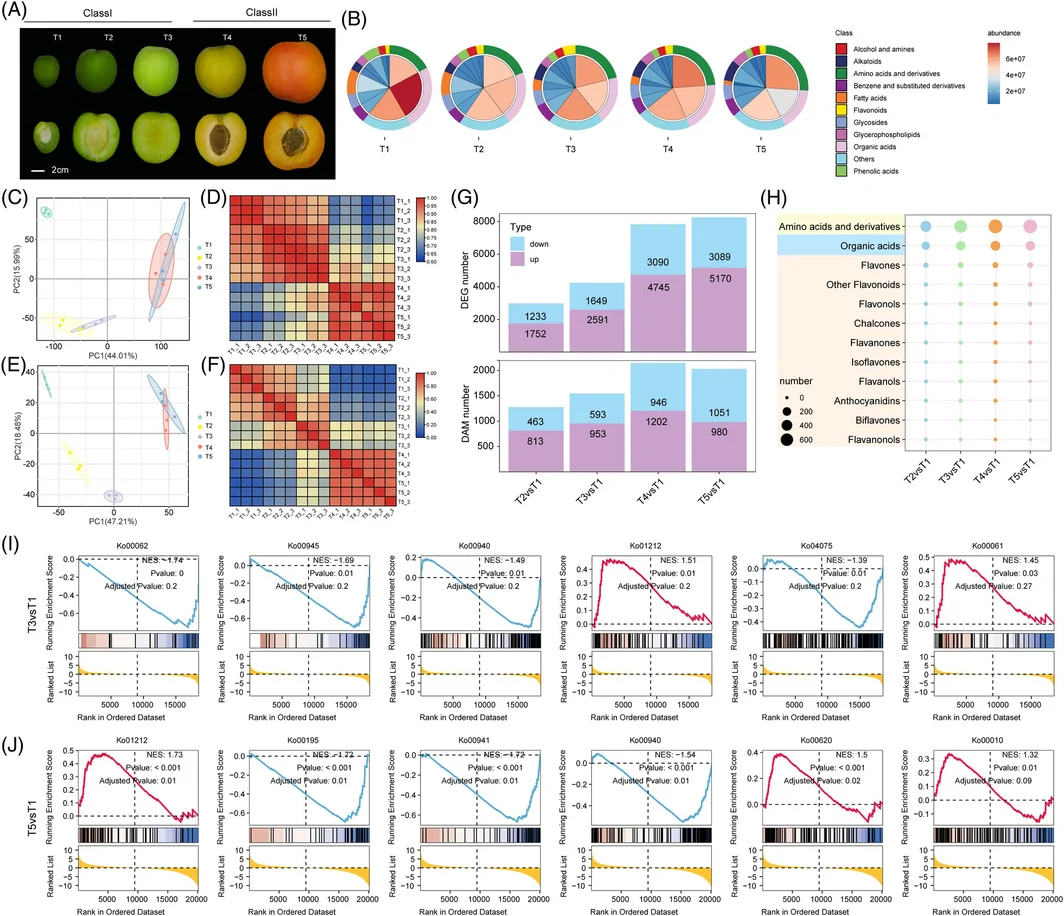
DEGs and DAMs increase during fruit development. (A) Picture of *P*. *armeniaca* Fyx fruits at 30 DPA (T1), 45 DPA (T2), 60 DPA (T3), 75 DPA (T4), and 80 DPA (T5). (B) Classification and number of annotated metabolites during apricot fruit development. (C–F) PCA and hierarchical clustering analyses of DEGs (C, D) and DAMs (E, F) based on transcriptomic and metabolomic data from T1 to T5. (G) Numbers of DEGs and DAMs during apricot fruit development. The bar charts show the numbers of upregulated and downregulated DEGs (upper panel) and up‐accumulated and down‐accumulated DAMs (lower panel) in pairwise comparisons between developmental stages (T2 versus T1, T3 versus T1, T4 versus T1, and T5 versus T1). (H) Classification of DAMs during apricot fruit development. Bubble plots represent the numbers of DAMs in different metabolite categories across pairwise developmental stage comparisons (T2 versus T1 to T5 versus T1). Bubble size indicates the number of metabolites in each category. (I, J) GSEA enrichment analysis of DEGs for T3 versus T1 (I) and T5 versus T1 (J). Gene sets were ranked by normalized enrichment score (NES), and significantly enriched pathways were identified using *P* < 0.05. Enrichment plots show running enrichment score (ES), gene set distribution, and gene positions along the ranked list.

To investigate metabolic dynamics and their regulatory mechanisms in apricot (*P. armeniaca* Fyx), flesh samples at five different stages (hereafter referred to as T1–T5) were collected, ranging from 30 to 80 days post‐anthesis (DPA). These samples can be categorized into Groups I and II based on phenotypic changes in flesh coloration during fruit development (Figure 3A). Group I fruits exhibited green flesh at 30, 45, and 60 DPA, whereas the flesh of fully ripe Group II fruits developed a yellow coloration at 75 and 80 DPA (Figure 3A). We then observed the changes in metabolites using broadly targeted LC–MS/MS‐based metabolic profiling. In total, 4292 metabolites were detected and annotated in apricot, including 1187 amino acids and derivatives, 532 organic acids, 478 benzene and substituted derivatives, 175 alkaloids, 175 flavonoids, 175 glycerophospholipids, 149 alcohol and amines, 145 phenolic acids, 143 heterocyclic compounds, 124 terpenoids, 105 nucleotides and derivatives, 101 lipids, and 803 other unclassified compounds (Figure S10; Table S22). Notably, amino acids and their derivatives generally accumulated during fruit development, whereas organic acids exhibited an overall declining trend with slight fluctuations from T1 to T5 (Figure 3B; Table S23).

We further conducted global transcriptomic analyses based on the same fruit samples using RNA‐seq. After quality filtering, an average of 57.76 million clean reads per sample was retained, yielding a total of 129.95 Gb of high‐quality data. The average data utilization rate reached 99.70%, with 97.05–98.87% of clean reads mapped to the apricot reference genome (Table S24). PCA and correlation analyses of metabolomic and transcriptomic data confirmed high reproducibility among three biological replicates and separated the samples into two distinct clusters corresponding to the five developmental stages (Figure 3C–F). Intriguingly, the clustering of metabolomic and transcriptomic profiles aligned with flesh color‐based phenotypic grouping of apricot across the five stages (Figure 3A). These findings suggest that metabolite accumulation parallels and correlates with global gene expression during apricot fruit development.

### Global metabolic and transcriptional dynamics during fruit development

**Figure 4:**
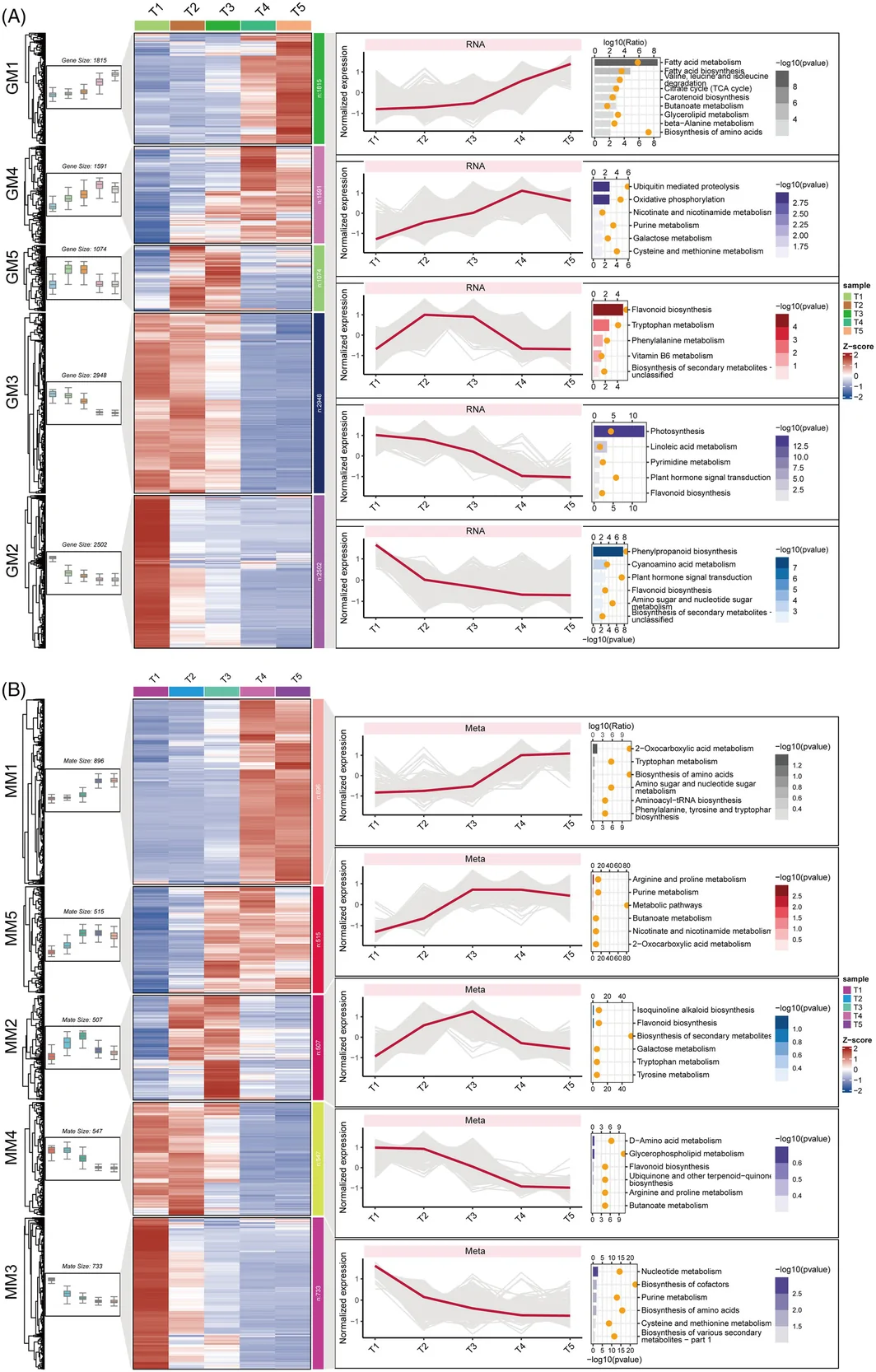
Metabolic dynamics of *Prunus armeniaca* Fyx at five developmental stages based on 9930 DEGs (A) and 3198 DAMs (B), respectively. Each cluster includes DEGs/DAMs statistics, heatmap, clustering analysis, and KEGG pathway enrichment.

To further elucidate the regulatory basis of metabolic changes during apricot development, both differentially accumulated metabolites (DAMs) and differentially expressed genes (DEGs) were subjected to mfuzz clustering analysis. Across four developmental comparisons (T2 versus T1, T3 versus T1, T4 versus T1, and T5 versus T1), we identified 3198 non‐redundant (NR) DAMs and 9930 NR DEGs, respectively (Figures S11 and S12). Both DAMs and DEGs exhibited a progressive and parallel increase from early (T2 versus T1) to late (T5 versus T1) stages, indicating tightly coupled metabolic and transcriptional reprogramming during fruit development (Figure 3G). Distinct patterns of metabolite accumulation were observed among the comparisons, with organic acids, amino acids, and their derivatives significantly enriched in T4 versus T1 and T5 versus T1 (Figure 3H). To explore the functional roles of genes within Groups I and II, a global test analysis based on gene set enrichment analysis (GSEA) was conducted across developmental transitions from the early (T3 versus T1) to late (T5 versus T1) stages. GSEA indicated a stage‐specific shift from T1 to T5, with fatty acid metabolism (ko01212) and fatty acid biosynthesis (ko00061) upregulated, while phenylpropanoid (ko00940) and flavonoid (ko00941) biosynthesis were downregulated, consistent with metabolite accumulation and flesh color changes (NES = 1.45–1.73 and − 1.72 to −1.49; *P* < 0.05; Figure 3I,J). Subsequently, mfuzz clustering partitioned these features into five distinct modules, each capturing characteristic temporal trajectories of metabolite accumulation and gene expression (Figure 4). Metabolic module I (MMI) was enriched in nucleotide metabolism with preferential accumulation at 30 DPA, whereas MMIII peaked at 60 DPA and comprised both alkaloid and flavonoids, followed by a gradual decline toward 80 DPA. In contrast, MMV displayed a marked increase from 75 to 80 DPA, largely driven by saccharides and amino acids (Figure 4A; Table S25). Like metabolite clustering, gene modules (GMs) mirrored the temporal trajectories of MMs based on their expression patterns, further supporting the coordinated dynamics between them throughout apricot fruit development (Figure 4B; Table S26).

### Accumulation of soluble sugars and organic acids in apricot fruits

**Figure 5:**
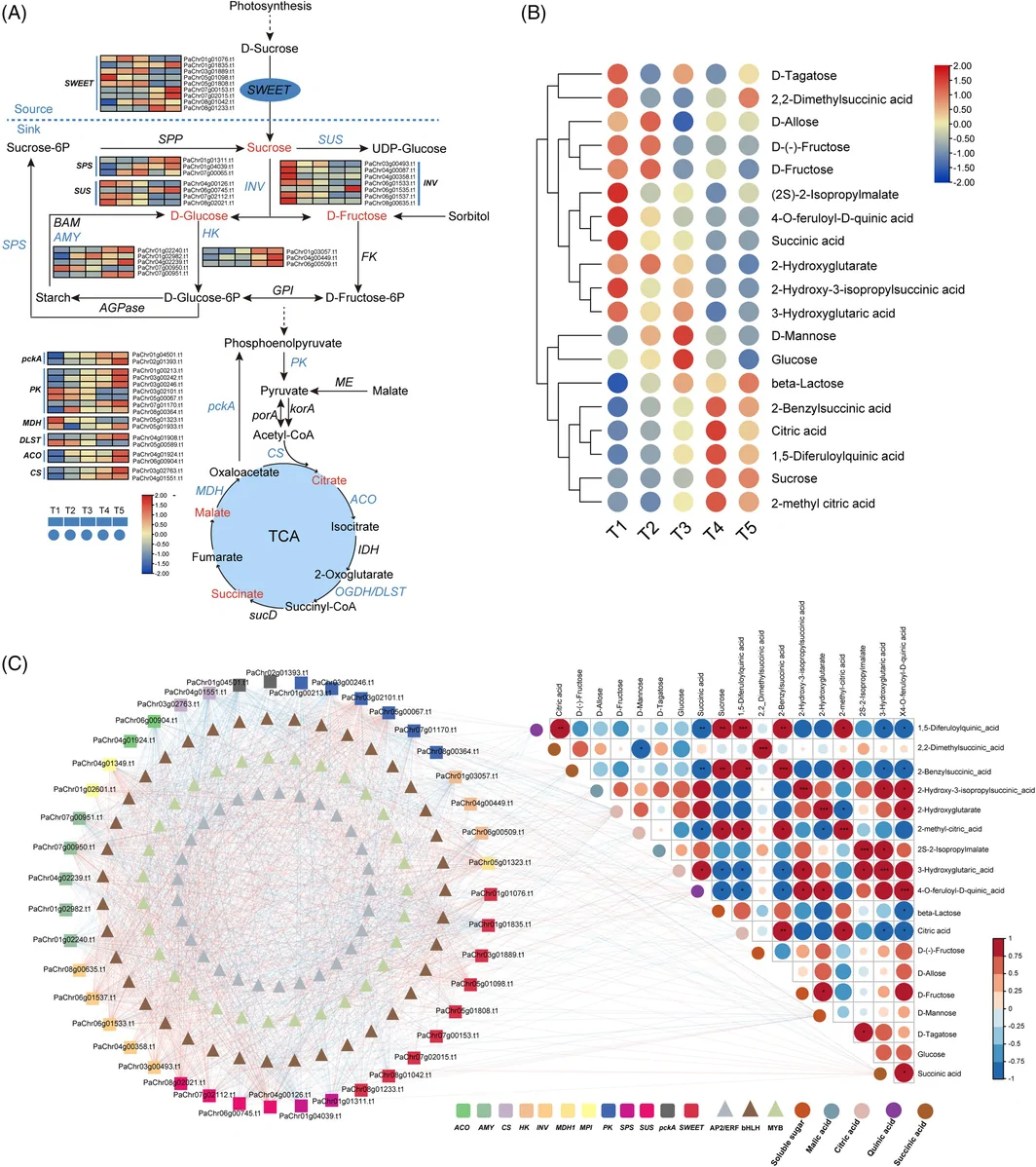
Identification of key genes of sugar and organic acid biosynthesis pathway in *Prunus armeniaca* Fyx. (A) Soluble sugar and organic acid biosynthesis pathway in Fyx. Square boxes indicate gene expression, and rounded boxes indicate metabolite accumulation. *ACO*, aconitate hydratase; *AMY*, alpha amylase; *CS*, citrate synthase; *DLST*, dihydrolipoamide S‐succinyltransferase; *HK*, hexokinase; *INV*, beta‐fructofuranosidase; *MDH*, malate dehydrogenase; *pckA*, phosphoenolpyruvate carboxykinase (ATP); *PK*, pyruvate kinase; *SPS*, sucrose phosphate synthase; *SUS*, sucrose synthase; *SWEET*, sugars will eventually be exported transporters. (B) Heat map of DAMs involved in sugar and organic acid biosynthesis. (C) The regulatory network of sugar and acid‐related metabolites in *Prunus armeniaca* Fyx. The triangle represents different transcription factors (TFs). The square represents structural genes involved in sugar and acid metabolism. The circles represent DAMs related to sugar and acid biosynthesis pathway. Pearson correlation coefficient values were calculated for each pair of metabolites, and the color scale from blue to red represents the correlation values from −1 to 1.

The sugar–acid ratio is a key determinant of fruit taste, quality, and harvest timing. In apricot, soluble sugars accumulation involves the starch and sucrose metabolism pathway, while the enrichment of organic acids is mainly related to glycolysis and the tricarboxylic acid (TCA) cycle (Fan et al., 2017). Targeting these pathways and mining the KEGG database, a total of eight saccharides were detected, including sucrose, glucose, D‐(‐)‐fructose, D‐fructose, D‐mannose, D‐allose, D‐tagatose, and β‐lactose. In addition, several organic acids were identified, comprising citric acid, 2‐methyl citric acid, 2‐hydroxyglutaric acid, 3‐hydroxyglutaric acid, and succinic acid, along with their derivatives, such as 2‐Hydroxy‐3‐isopropylsuccinic acid, (2S)‐2‐Isopropylmalate, 4‐O‐feruloyl‐D‐quinic acid, 1,5‐Diferuloylquinic acid, and 2,2‐Dimethylsuccinic acid (Table S27). Among these metabolites, sucrose and citric acid accumulated to the highest levels at T4, whereas glucose, D‐(−)‐fructose, D‐fructose, and succinic acid declined significantly during this stage (Figure 5A,B). Expression of genes encoding α‐amylase (*PaAMY*; 3/5), hexokinase (*PaHK*; 3/3), and sucrose phosphate synthase (*PaSPS*; 2/3) genes was markedly upregulated at T4 and T5. Similarly, key citric acid metabolism genes, including citrate synthase (*PaCS*) and aconitase (*PaACO*), showed peak expression at the same stages, indicating coordinated regulation of carbohydrate and organic acid metabolism during fruit maturation (Table S28). In addition, we also discovered a series of TFs involved in the sugar and acid synthesis pathway in apricot, such as AP2/ERF, bHLH, and MYB. The potential regulatory network mediated by these TFs was visualized in Figure 5(C). We found that there was high connectivity between these TFs and many fruit ripening‐associated genes, such as *PaCS* (PaChr03g02763.t1, PaChr04g01551.t1), involved in citric acid biosynthesis, and *PaSUS* (PaChr04g00126.t1, PaChr06g00745.t1), *PaINV* (PaChr06g01533.t1, PaChr06g01537.t1), and *PaAMY* (PaChr01g02240.t1, PaChr07g00950.t1) participating in sugar and starch metabolism.

### DNA methylation increases during apricot fruit development

**Figure 6:**
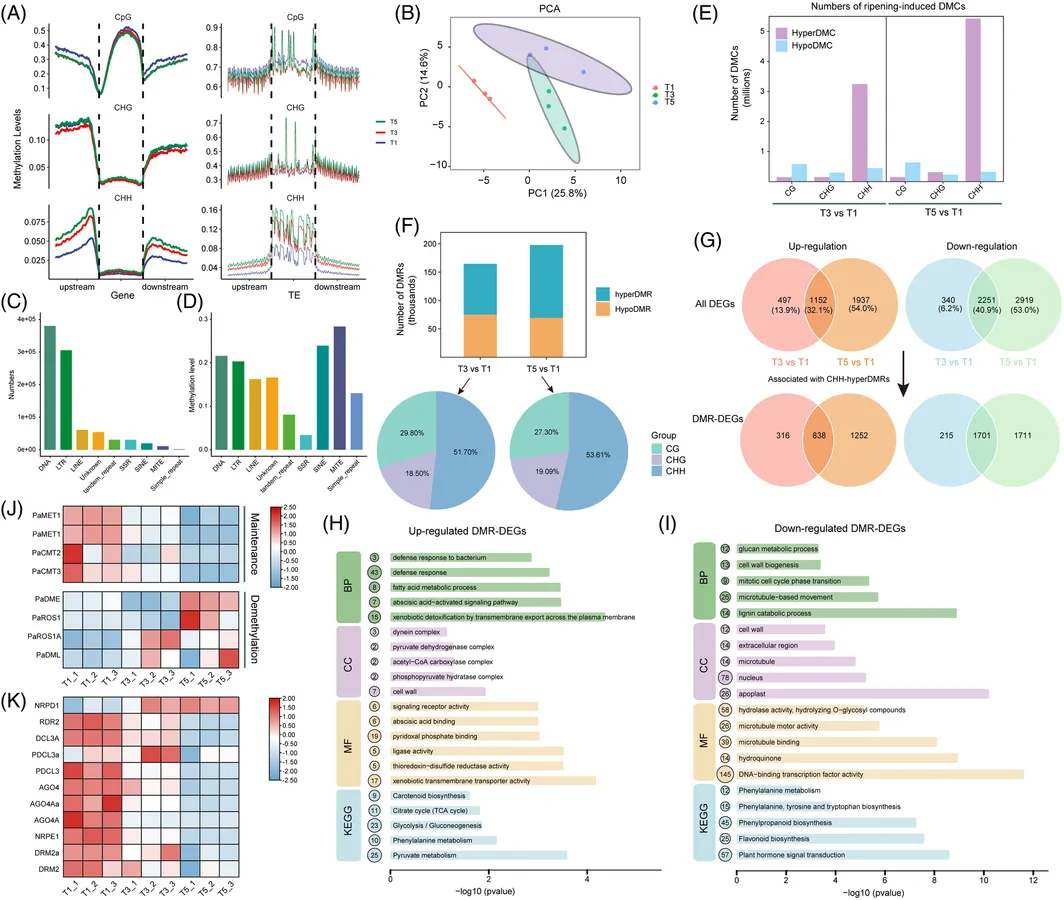
Genome‐wide increase in DNA methylation during *Prunus armeniaca* Fyx fruit development and ripening. (A) DNA methylation levels of genes and TEs in T1–T5. Methylation levels of mC, mCG, mCHG, and mCHH are shown. (B) PCA showing consistency between the three biological replicates of whole‐genome bisulfite sequencing samples of T1–T5. (C) Number of the nine representative types of TEs in the apricot genome. (D) The average DNA methylation levels of the nine TE types. (E) Numbers of ripening‐induced DMCs in T5 relative to T1 are shown for the mCG, mCHG, and mCHH sequence contexts. (F) Numbers of hyper‐ and hypo‐DMRs in T3 and T5 relative to T1. (G) Comparisons of DEGs and DMRs of T3‐T1 and T5‐T1 groups, respectively. The Venn diagram demonstrates the number of overlapping DEGs and DMR‐associated DEGs of T3‐T1 and T5‐T1 groups. (H, I) The barplot showing GO and KEGG enrichment analysis of upregulated (H) and downregulated (I) DEGs associated with CHH‐hyper‐DMRs. (J, K) The heatmap showing the expression changes of representative genes encoding DNA methylase, demethylase (J) and pivotal genes involved in the RdDM pathway of three fruit development stages (K).

To explore the relationship between DNA methylation and gene expression, we generated single‐base resolution maps of apricot fruit at three different stages, T1, T3, and T5, corresponding to critical phases of fruit development (Figures 1A and 6A). Each stage was sequenced with three biological replicates, generating at least 41.58 million reads per replicate and achieving >24× genome coverage, with ≥54.15% uniquely mapped reads and ≥99.67% coverage of genomic cytosines (Table S29). Correlation analysis (Figure S13) and PCA (Figure 6B) demonstrated high consistency among biological replicates. Bisulfite conversion efficiency exceeded 99%, indicating that the sequencing quality and depth were sufficient for downstream analyses. We assessed genome‐wide DNA methylation in apricot fruits and found that CG and CHG sequence contexts exhibited similar patterns, with methylation levels gradually decreasing during development. In contrast, CHH methylation followed a distinct trajectory, initially increasing and then slightly decreasing (25.96% at T1, 28.68% at T3, and 30.01% at T5; Table S30). We next examined the average methylation levels of genes and TEs across developmental stages. From T1 to T5, DNA methylation gradually increased in the 5′ and 3′ flanking regions of genes, as well as within TE bodies and their flanking regions (Figure 6A). All TEs were classified into nine subgroups, with DNA and LTR representing the major fractions (Figure 6C). Most subgroups exhibited high methylation levels except for the TRs and simple sequence repeats (SSRs) (Figure 6D). Comparison of the DNA methylomes between T1 and T5 identified 7.07 million differentially methylated cytosines (DMCs), of which 83.24% were hypermethylated (hyper‐DMCs) and 92.15% of these occurred in the CHH context, suggesting that CHH sites largely account for the observed increase in DMCs during development (Figure 6E). To further characterize differentially methylated regions (DMRs), we compared the methylomes of T3 and T5 with that of T1, respectively. As shown in Figure 6(F), the number of hyper‐DMRs relative to T1 gradually increased from 89 673 in T3 to 128 356 in T5 (Chi‐square test, *P* < 2.2e‐16), consistent with a global increase in DNA methylation during fruit development.

### Correlation of DNA methylation with gene expression changes

To investigate the effect of DNA methylation on gene expression in the CHH context, differential methylation genes (DMGs) were defined as genes together with their 2 kb upstream and downstream regions overlapping with DMRs. Compared with T1, 3586 and 5510 genes were upregulated and downregulated in T3 and T5, respectively (|Log2FC| > 1, *P* < 0.05), of which 32.10% (1152) and 40.9% (2251) were shared between T3 and T5 (Figure 6G). Notably, 66.30% of DEGs (6033 in DMGs/9096 in all DEGs) were classified as DMGs (hypergeometric test, *P* = 2.77e‐7), suggesting that differential expression preferentially occurs in genes with methylation changes and highlighting the potential role of DNA methylation in the regulation of gene expression during apricot development. Furthermore, GO and KEGG enrichment analyses were conducted on the upregulated (838) and downregulated (1701) DEGs overlapping with DMGs. These genes were significantly enriched in pathways associated with key apricot phenotypes, including phenylalanine metabolism, phenylpropanoid biosynthesis, flavonoid biosynthesis, and the citrate cycle (TCA cycle), as well as biological processes essential for plant growth and development (Figure 6H,I).

### Increased expression of RdDM‐related genes during apricot development

Cytosine methyltransferases and demethylases are key regulators of DNA methylation dynamics, governing diverse biological processes in plants (Stroud et al., 2014; Zhu et al., 2007). Here, *PaMET1* (for CG) and *PaCMT2/PaCMT3* (for CHG), responsible for maintenance methylation, were highly expressed at T1 than at T3 and T5 stages, consistent with the decline in CG and CHG methylation during apricot development.

The demethylases *Pa*DME, *PaROS1*, *PaROS1A*, and *PaDML* exhibited expression trends opposite to the methyltransferases (Figure 6J; Table S31), consistent with the methylation variation, suggesting that CG and CHG methylation levels are coordinately regulated by both methyltransferases and demethylases. By contrast, the CHH methylation pattern was uncoupled from methyltransferase and demethylase expression, owing to its context‐specific regulation through *de novo* methylation mediated by the RdDM pathway. Correspondingly, we identified key genes involved in the RdDM pathway and analyzed their expression profiles in apricot. With the exception of *PaNRPD1* and *PaPDCL3*, six RdDM‐associated genes (*PaRDR2*, *PaDCL3A*, *PaAGO4A*, *PaNRPE1*, *PaDRM2*, *PaDRM2*) displayed higher expression at T1 than at T3 and T5, in contrast to the genome‐wide CHH methylation pattern (Figure 6K; Table S31), indicating a temporal uncoupling between RdDM gene expression and CHH methylation establishment. Thus, methylation changes in the CHH context during apricot development are likely associated with the RdDM pathway.

### DNA methylation regulates anthocyanin biosynthesis

**Figure 7:**
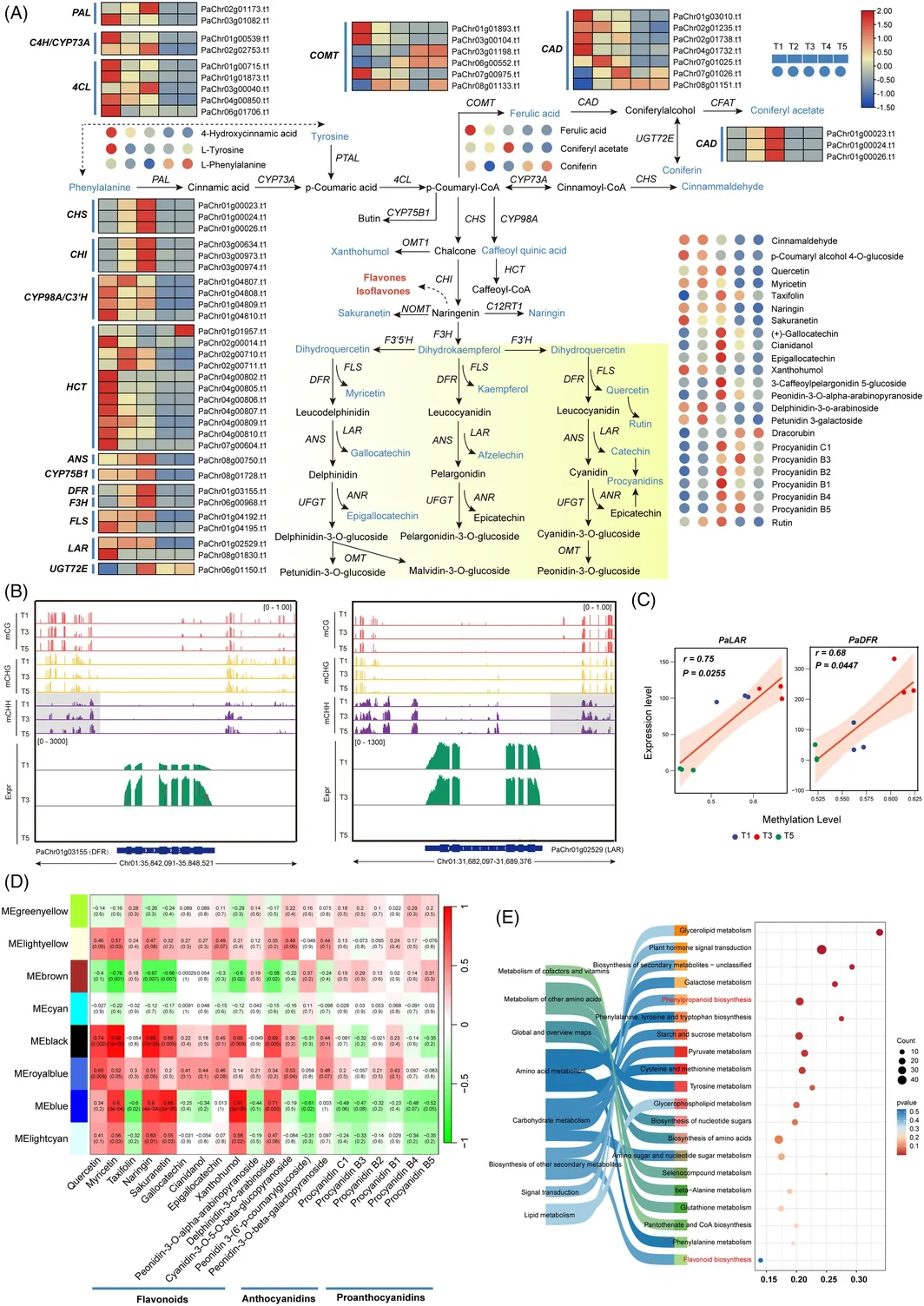
Spatial expression patterns and anthocyanin biosynthesis in *Prunus armeniaca* Fyx. (A) Anthocyanin biosynthesis pathway in Fyx. Square boxes indicate gene expression, and rounded boxes indicate metabolite accumulation. *4CL*, 4‐coumarate‐‐CoA ligase; *ANS*, anthocyanidin synthase; *C4H/CYP73A*, *trans*‐cinnamate 4‐monooxygenase; *CAD*, cinnamyl‐alcohol dehydrogenase; *CHI*, chalcone isomerase; *CHS*, chalcone synthase; *COMT*, caffeic acid 3‐O‐methyltransferase; *CYP75B1*, flavonoid 3′‐monooxygenase; *CYP98A*, C3′H, 5‐O‐(4‐coumaroyl)‐D‐quinate 3′‐monooxygenase; *DFR*, bifunctional dihydroflavonol 4‐reductase/flavanone 4‐reductase; *F3H*, naringenin 3‐dioxygenase; *FLS*, flavonol synthase; *HCT*, shikimate O‐hydroxycinnamoyltransferase; *LAR*, leucoanthocyanidin reductase; *PAL*, phenylalanine ammonia lyase; *UGT72E*, coniferyl‐alcohol glucosyltransferase. (B) Genome browser view of DNA methylation and expression levels of *PaLAR* and *PaDFR*. The DNA methylation levels are represented by the height of the vertical bars in each track (CG: red; CHG: yellow; CHH: purple). Gray shaded areas indicate the locations of DMRs. (C) Correlation between promoter DNA methylation (2 kb upstream) and expression of *PaLAR* and *PaDFR* during apricot fruit ripening. (D) Co‐expression modules identified by WGCNA and their associations with hub DAMs in Fyx. The heat map shows Pearson correlation coefficients (*r*) between modules and hub DAMs, with r (upper) and *P*‐values (lower) indicated in each cell. (E) KEGG enrichment analysis of genes in the MEblue module.

In apricot, the T4–T5 stage marks the transition from nearly ripe to fully mature fruit, with anthocyanin biosynthesis driving the associated color changes (Figure 3A). The red flavonoid metabolites identified were mainly composed of five anthocyanins, including petunidin 3‐galactoside, delphinidin‐3‐o‐arabinoside, peonidin‐3‐O‐alpha‐arabinopyranoside, 3‐Caffeoylpelargonidin 5‐glucoside, and dracorubin, along with seven proanthocyanidins (PAs) (Figure 7A; Table S32). These metabolites generally accumulated at higher levels during the early to middle stages of fruit development (T1–T3), similar to other flavonoid compounds such as chlorogenic acid, ferulic acid, coniferin, quercetin, naringin, and xanthohumol. Notably, the accumulation of dracorubin, a red flavonoid derivative, increased during the later stages, consistent with changes in fruit flesh coloration (Figures 3A and 7A; Table S32). We examined the expression patterns of essential genes in flavonoid biosynthetic pathway to gain insight into anthocyanin/PA accumulation. The results indicated that a series of genes were substantially upregulated during early fruit development, consistent with the increased metabolite accumulation from T1 to T3. For example, genes such as chalcone synthase (*PaCHS*; 3/3), chalcone isomerase (*PaCHI*; 3/3) and flavanone 3‐hydroxylase (*PaF3H*; 1/1) increase slightly in the T3 stage but decrease in the T4–T5 stage. In contrast, *PaDFR*, flavonoid‐3′,5′‐hydroxylase (*PaF3′5′H*) and anthocyanidin synthase (*PaANS*) showed a rapid up expression at T3 stage (Table S33; Figure 7A). In PA biosynthesis, catechin and epicatechin are derived from leucocyanidin and cyanidin, respectively, and these conversions are catalyzed by *PaLAR* (PaChr01g02529.t1) and *PaDFR* (PaChr01g03155.t1). Notably, we observed a significantly higher level of CHH methylation in the promoter subregions of *PaDFR* and *PaLAR* from stage T1 to stage T3 (Figure 7B; Table S34). Specifically, hyper‐DMRs were found in the 1–2 kb promoter of *PaLAR*, as well as the ≤1 kb and 2–3 kb promoter segments of *PaDFR*. Multiple core promoter elements and TF binding motifs, including TATA‐box, CAAT‐box, MYB, MYC, and CGTCA motif, were predicted within these DMRs (Table S34). This elevated methylation was positively associated with *PaDFR* and *PaLAR* expression (Figure 7C), suggesting a potential role of CHH methylation in regulating gene transcription during apricot development.

### Novel TFs in regulation of proanthocyanidin metabolism

To further explore the correlation between anthocyanin levels and potential hub regulatory genes, a weighted gene correlation network analysis (WGCNA) was performed using 7831 genes (with an average FPKM >0.5) expressed in 15 apricot fruit samples. A total of eight co‐expression modules were identified based on similar expression patterns. The module–trait correlation heatmap revealed a strong positive correlation (*R* > 0.8) between genes in the MEblue module and the accumulation of key flavonoid‐related metabolites (Figure 7D; Figure S14). We further characterized the distribution and functional features of DMRs in the MEblue module and found that DMRs were preferentially located in gene promoters and distal intergenic (Fisher's exact test, *P* < 1.0e‐5) (Figure S15A). Specifically, both hyper‐DMRs and hypo‐DMRs were mainly distributed in gene bodies. Hyper‐DMRs showed higher signal intensity near the 5′ ends in T1 versus T3 than in T1 versus T5, whereas hypo‐DMRs exhibited the opposite trend (Figure S15B,C). Hyper‐DMRs were also preferentially located at the 5′ ends of TEs, with consistent distribution patterns between T1 versus T3 and T1 versus T5 (Figure S15D,E). KEGG enrichment analysis of genes in the MEblue module highlighted phenylpropanoid and aromatic amino acid (phenylalanine, tyrosine, tryptophan) biosynthesis pathways, all of which are implicated in flavonoid production (Figure 7E; Table S35).

**Figure 8:**
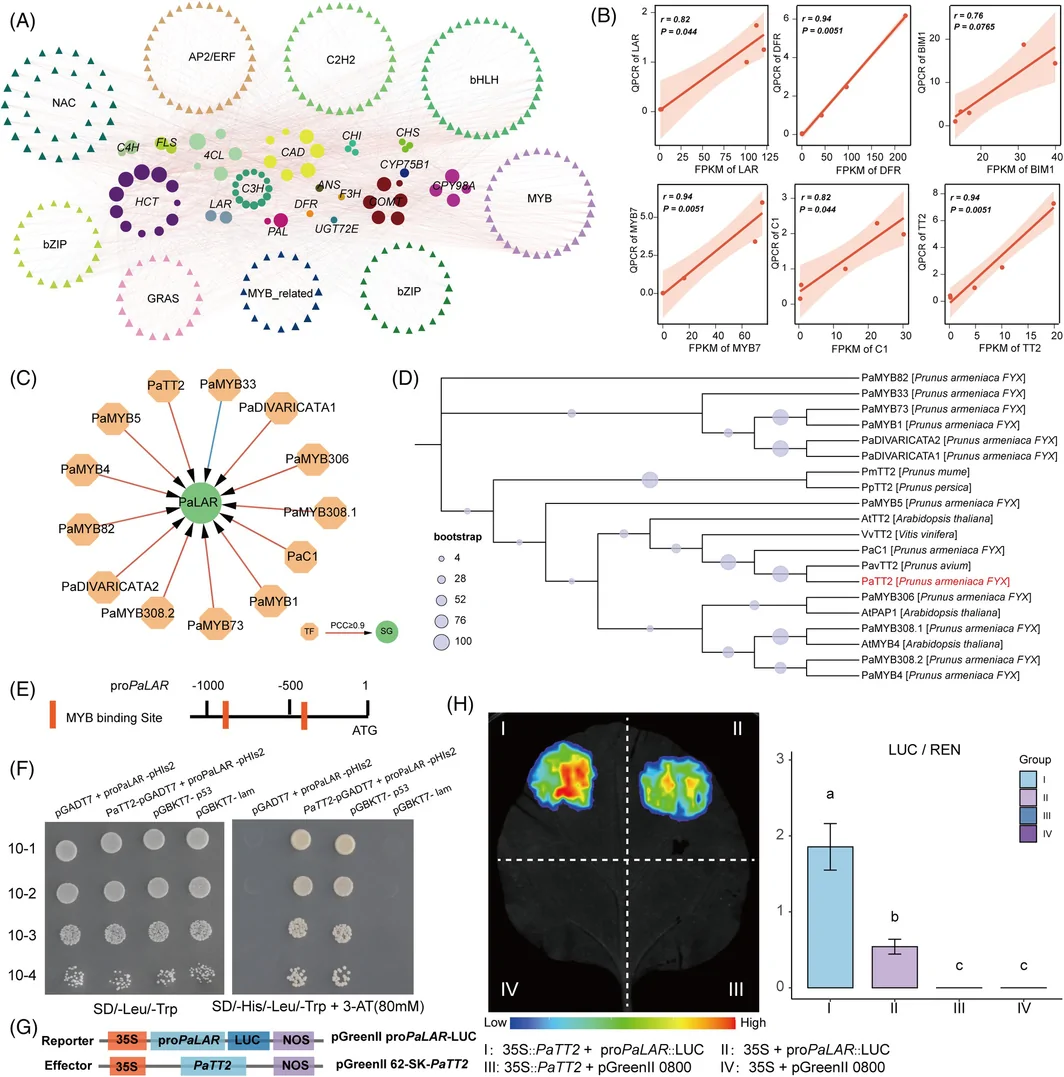
*PaTT2* directly binds to the promoter of *PaLAR*. (A) Visualization of the co‐expression gene and TF network related to key anthocyanin biosynthesis pathways in *Prunus armeniaca* Fyx. Triangles represent TFs, and squares represent DEGs involved in anthocyanin biosynthesis. (B) Correlation between qRT‐PCR and gene expression levels. (C) MYB‐associated subnetwork in proanthocyanidin metabolic regulatory network. (D) Phylogenetic tree of MYB proteins from *P. armeniaca* Fyx and other species. (E) Schematic representation of the promoter region of *PaLAR*. (F) The binding of *PaTT2* to the *PaLAR* promoter examined using a yeast one‐hybrid assay. Yeast cells were co‐transformed with pGBKT7‐*PaTT2* and pGADT7‐proLAR‐pHIS2 vectors and grown on selective media SD/‐Trp/‐Leu/‐His +3‐AT (80 mM). (G) Schematic illustration of effector and reporter constructs vectors used for transient transcriptional activity assays in tobacco leaves. (H) Representative images of tobacco leaves 48 h after infiltration and relative firefly luciferase activity normalized to Renilla luciferase activity. Enhanced GUS staining was observed in leaves co‐transformed with *PaTT2* and the *PaLAR* promoter. LUC/REN ratios (y‐axe) are shown for different co‐infiltration combinations. Data are presented as mean ± SD with three biological replicates. Different lowercase letters indicate significant differences based on Tukey's HSD test (*P* < 0.05). Asterisks indicate significance based on Student's *t*‐test (*P < 0.05; **P < 0.01; ns, not significant).

TFs from the bHLH, MYB, AP2/ERF, and MYB‐related families are well known to regulate PA metabolism in Arabidopsis, rice, apple, and other plants (Baudry et al., 2004; Dubos et al., 2008; Furukawa et al., 2007; Shu et al., 2023; Zhang et al., 2022). To characterize the TFs that putatively regulate anthocyanins, we constructed a potential regulatory network consisting of 44 hub genes highly correlated with flavonoids (|*R*| ≥ 0.9, *P* < 0.05) and their upstream regulatory TFs in the MEblue module (Figure 8A). We found that the network comprised 303 nodes and 3081 edges, with an average connectivity of 20.85 neighbors per node, and bHLH, MYB, MYB‐related, and AP2/ERF were the major TF families potentially regulating anthocyanin metabolism in apricot. To validate the transcriptome data, the expression patterns of the six genes of qRT‐PCR across different fruit developmental stages showed strong agreement with the RNA‐seq (Figure 8B). As MYB TFs are widely recognized as core regulators of PA biosynthesis that activate or repress target genes via promoter binding (e.g., Baudry et al., 2004; Dubos et al., 2008; Furukawa et al., 2007; Schaart et al., 2013), we specifically focused on the MYB‐associated subnetwork (Figure 8C). Notably, *PaC1* (*PaChr01g03397.t1*, PCC = 0.94, *P*‐value = 0.01), *PaTT2* (*PaChr04g00308.t1*, PCC = 0.92, *P*‐value = 0.002) and *PaMYB1* (*PaChr02g00885.t1*, PCC = 0.91, *P*‐value = 0.03) were strongly co‐expressed with *PaLAR*. Phylogenetic analysis pinpointed *PaTT2* (PaChr04g00308.t1) as a MYB candidate (Figure 8D). This gene is orthologous to *PavTT2* (*Prunus avium*), *VvTT2* and *AtTT2*, and only *AtTT2* has been functionally validated as a master regulator of PA accumulation in Arabidopsis (Baudry et al., 2004). Similarly, CHH methylation levels in the promoter regions of *PaTT2* were significantly higher from stage T1 to stage T3 (Figure S16; Table S34). To assess the functional relevance of candidate MYB genes, we analyzed the *PaLAR* promoter using PlantCARE and discovered that the 1 kb position of the promoter contained multiple MYB‐binding elements (Figure 8E), which might be the binding sites of *PaLAR* to regulate PA metabolism.

To further confirm *PaTT2* directly binds to and activates the transcription of *PaLAR* in *P. armeniaca*, yeast one‐hybrid (Y1H) and dual‐luciferase reporter assays were performed. In the Y1H experiments, compared with the positive and negative controls, the yeast cells simultaneously transformed with both the pGADT7‐His2::pro*PaLAR* and pGBKT7::PaTT2 constructs were capable of growing on the SD/‐His/‐Leu/‐Trp/ medium added to 60 mM 3‐AT (Figure 8F). Subsequently, dual‐luciferase reporter assays were employed to assess the transcriptional regulatory effects of *PaTT2* on the *PaLAR* promoter (Figure 8G). Compared with the empty vector transformation control, co‐transformation of the effector (pGreenII‐62‐SK‐*PaTT2*) with the reporter constructs (pGreenII‐pro*PaLAR*‐LUC), a significantly increased LUC signal was observed in tobacco leaves in the dual‐luciferase reporter assay (Figure 8H). Taken together, these results indicate that *PaTT2* possibly binds to the *PaLAR* promoter and activates its transcription, thereby promoting PA biosynthesis in apricot.

## DISCUSSION

A complete reference genome is essential for investigating genetic diversity and adaptive evolution in horticultural species. Nevertheless, previously released genome assemblies of apricot still exhibit varying degrees of assembly gaps and lack genetic information in centromeric and telomeric regions. This limitation has constrained investigations into how genetic variations in these regions influence key agronomic traits. Sometimes, agriculturally important loci may reside within these inaccessible regions. For instance, the gene positively regulating tiller number in rice was identified within the centromeric region of chromosome 12, while quantitative trait loci (QTLs) associated with berry color and sex determination were mapped to chromosome 2 in grapevine (Fournier‐Level et al., 2009; Lv et al., 2024; Zou et al., 2021). Consequently, a gapless reference genome holds significant potential for elucidating the genetic basis of polygenic agronomic traits and unraveling mechanisms underlying species evolution and artificial selection. This is, to the best of our knowledge, the first T2T gap‐free genome assembly in apricot. The average N50 value of this genome is 30.64 Mb, which is 3.9 M higher than previously published genomes. The T2T genome assembly in this study filled all the gaps in previous assemblies and completed the assembly of centromeres and telomeres, which allows us to gain comprehensive characterization of structurally complex regions including TRs, retrotransposon arrays, centromeres, and telomeres that drive genomic innovation and species divergence. Moreover, the T2T version could also be used as robust genetic resources for apricot functional genomics and breeding research.

The spatial distribution of TEs and SRFs relative to centromeric domains revealed significant positional biases for specific repeat superfamilies, exhibiting varying degrees of correlation or anticorrelation with proximity to centromeric cores, is similarly conserved in the grape genome (Shi et al., 2023). These centromeric repeat sequences transcribe into non‐coding RNAs that maintain centromere structure and ensure accurate chromosome segregation by forming specific structures or binding to histones (Liu et al., 2021). The detection of structural variations enhanced our understanding of genomic architecture and evolutionary relationships both within and among these *Prunus* species, underscoring the critical role of genomic rearrangements in speciation and adaptation. However, compared with the other three genomes, there were more significant genetic variations between the genomes of *P*. *armeniaca* Fyx and *P*. *armeniaca*, and Fyx and *P*. *mume*, such as inversions and translocations. A plausible explanation is that *P*. *armeniaca* and *P*. *mume* may have undergone more intensive artificial selection within *Prunus* species, thereby driving this divergence. Additionally, the SD events detected in the *P*. *armeniaca* genome may have contributed to the elevated SVs between its chromosomes and those of *P*. *armeniaca* Fyx. Nevertheless, whether these structural variation regions are closely associated with fruit flavor traits or adaptive evolution in plants may require further investigation.

Gene duplication serves as a fundamental driver of functional diversification in protein‐coding genes, promoting divergence and the adaptive evolution of close relative species (Wei et al., 2021; Xiang et al., 2017). Phylogenomic analyses revealed that *P*. *armeniaca* Fyx underwent an ancient WGD event shared by other Rosaceae lineages, which likely facilitated adaptive radiation and species diversity through subfunctionalization of conserved gene networks (Liu et al., 2020; Zhou et al., 2024). Furthermore, a recent SD event unique to *P*. *armeniaca* Fyx, distinct from the genomic architecture of *P*. *armeniaca*, probably serves as a primary driver of interspecific divergence, generating variations in genome size and genetic composition (Zhao et al., 2017). CAFE analyses revealed that 281 gene families underwent expansion and 634 contracted in *P*. *armeniaca* Fyx during its evolution. The significant enrichment of expanded gene families in ubiquitin‐dependent protein catabolic process, ADP binding, polysaccharide binding, and ubiquitin‐mediated proteolysis highlights critical adaptive mechanisms in *P*. *armeniaca* Fyx. Defense‐related expansions enhance resistance to biotic stressors by facilitating pathogen recognition and triggering hypersensitive responses (Zhang et al., 2024). Ubiquitin–proteasome system (UPS) components regulate hormone signaling and protein turnover, enabling precise control of developmental transitions and stress responses (Kurepa & Smalle, 2008). For instance, UPS‐mediated degradation fine‐tunes floral organ identity genes in ginger, affecting inflorescence development (Xing et al., 2021). ADP/polysaccharide‐binding domains in enzymes like UDP‐glycosyltransferases (UGTs) modify secondary metabolites (e.g., flavonoids, terpenoids), influencing fruit quality and stress adaptation in tomato (Huang et al., 2025). Concurrently, ubiquitin‐mediated proteolysis and autophagy collaborate to remove damaged proteins under stress, maintaining cellular homeostasis. Thus, these significantly enriched expanded genes might be functionally associated with inflorescence development, fruit quality, and stress adaptation in *P*. *armeniaca* Fyx. The contraction of gene families enriched in carbohydrate metabolism and phenylpropanoid biosynthesis reflects adaptive optimization in *P*. *armeniaca* Fyx for energy conservation and stress resilience. Reduced carbohydrate metabolic genes may mitigate carbon depletion during dormancy under warming winters, while diminished phenylpropanoid biosynthesis could reallocate resources from secondary metabolites (e.g., flavonoids) to essential defense pathways (Brukental et al., 2021; Ortuño‐Hernández et al., 2024). ABC transporter contraction may impair hormone (e.g., auxin) and xenobiotic transport, affecting stress responses (Liu & Liao, 2025). Enhanced hydrogen peroxide catabolism and oxidative stress response likely maintain redox homeostasis, crucial for drought tolerance. Pyruvate metabolism alterations may optimize energy flux from glycolysis to the Krebs cycle, supporting survival in nutrient‐limited habitats (Ortuño‐Hernández et al., 2024). These genomic adjustments align with ecological strategies to prioritize core physiological functions over expendable pathways.

Although advanced metabolite profiling techniques have identified numerous compounds associated with fruit quality, the precise regulatory networks governing their accumulation remain unclear. Supported by the high‐quality *P. armeniaca* Fyx genome, integrated transcriptomic and metabolomic analyses revealed stage‐specific dynamics of soluble sugars, organic acids, and other nutritional metabolites, characterized by two major transitions (T1–T3 and T4–T5) that reflect coordinated metabolic reprogramming during fruit maturation. These transitions parallel metabolic trajectories reported in other fruits, such as kiwifruit (Shu et al., 2023; Zeng et al., 2024) and mango (Wu et al., 2022). Notably, the shift from the nearly ripe to the fully mature stage appears to be critical for sucrose and citric acid biosynthesis, with the highly expressed *PaAMY*, *PaSPS*, *PaPK*, *PaCS*, and *PaACO* likely serving as key enzymatic drivers of metabolite accumulation (Figure 5). Furthermore, the TF landscape during apricot maturation indicates that bHLH, MYB, and AP2/ERF families are likely involved in the coordination of sugar and organic acid metabolism. bHLH members were the most abundant and showed strong correlations with sugar‐ and acid‐metabolism genes, consistent with their established roles in regulating developmental and ripening processes in fleshy fruits (Shi et al., 2025; Zhang et al., 2020). Selected MYB factors also exhibited stage‐dependent expression and association with major metabolites, which aligns with the broad regulatory functions of the MYB family in primary and secondary metabolism (Dubos et al., 2010). In addition, AP2/ERF TFs, central components of ethylene‐responsive regulatory networks, were linked to late‐stage metabolic transitions, in agreement with their established roles in climacteric fruit ripening, including in *Prunus* species (Liu et al. 2015; Zeng et al., 2025). These results collectively suggest that multiple TF families act in concert to modulate sugar–acid homeostasis during ripening. Nevertheless, verifying their direct regulatory functions will require promoter‐binding assays and targeted functional studies.

DNA methylation, alongside hormonal signals and TFs, critically regulates ripening in fruit crops, exhibiting species‐specific dynamics and regulatory patterns, as demonstrated in tomato (Lang et al., 2017; Zhong et al., 2013), sweet orange (Cheng et al., 2018; Huang et al., 2019), peach (Zhu et al., 2020), strawberry (Li et al., 2023), lemon (Yu et al., 2024), grape (Wei et al., 2024), and pear (Gu et al., 2024). In the present study, using whole‐genome bisulfite sequencing, we found that the dynamics of DNA methylation during apricot fruit ripening was completely different from that of tomato (Lang et al., 2017). Specifically, CHH hypermethylation occurred in the absence of CG or CHG hypomethylation, driving an overall increase in genome‐wide methylation levels (Figure 6). By contrast, in tomato, global CG and CHG hypomethylation occurs concomitantly with CHH hypermethylation during ripening (Lang et al., 2017). Notably, we found that DNA methylation globally increases during apricot ripening, consistent with observations in sweet orange and lemon (Huang et al., 2019; Yu et al., 2024). These methylation dynamics suggest that the regulation of DNA methylation is crucial for normal fruit ripening, even though the direction of methylation changes differs between species (Huang et al., 2019). Moreover, DNA demethylase genes were upregulated during apricot fruit ripening, whereas DNA methyltransferase genes were relatively high abundant but downregulated (Figure 6). These results suggest that dynamic changes in DNA methylation during fruit ripening are co‐regulated by both methyltransferase‐ and demethylase‐mediated activities. Interestingly, the observed increase in CHH methylation levels is likely attributable to a reduction in RdDM pathway activity, as several key RdDM components were significantly downregulated during apricot fruit ripening (Figure 6). Taken together, our findings reveal a dynamic remodeling of DNA methylation during apricot fruit ripening, providing new insights into the epigenetic mechanisms underlying this process.

DNA methylation has been characterized to regulate anthocyanin biosynthesis during fruit development, as observed in peach (Zhu et al., 2020) and pear (Gu et al., 2024). In apricot, most PAs accumulated from the early (T1) to middle (T3) stages, followed by a decline toward the late (T5) stage, corresponding with significant upregulation of *PaDFR* and *PaLAR* (Figure 7). *DFR* and *LAR* function as key enzymes in PA biosynthesis, catalyzing the conversion of dihydroflavonols to leucoanthocyanidins and subsequently to catechin‐type flavan‐3‐ols, respectively (Dixon & Sarnala, 2020), and have been shown to contribute to anthocyanin biosynthesis in various fruits such as crabapple, grape, and banana (Li et al., 2019; Rajput et al., 2022; Yu et al., 2019). Our study further reveals that high expression of *PaDFR* and *PaLAR* may be associated with elevated CHH methylation in their promoters (Figure 6B), suggesting that DNA methylation modulates PA accumulation. Anthocyanin and PA biosynthesis are close branches belonged to flavonoid pathway, which are generally dynamically balanced in plants (Li et al., 2025). Furthermore, we identified *PaTT2* as a MYB TF regulates PA‐related gene *PaLAR*, thereby broadening our understanding of PA regulatory mechanisms and providing a potential target for gene‐editing‐mediated color breeding (Figure 8). This finding aligns with previous reports that MYB regulators control anthocyanin/PA pathways in a tissue‐specific manner in several fruit species (Jiang et al., 2023; Karppinen et al., 2021; Yu et al., 2023). Consequently, DNA methylation and TFs likely contribute to anthocyanin synthesis by modulating the expression of key genes in the flavonoid pathway, although the underlying molecular mechanisms require further investigation.

## MATERIAL AND METHODS

### Plant materials and genome sequencing

“Fengyuanxing” apricots were sampled from a commercial orchard in Huyi District, Shaanxi Province, China (34.05° N, 108.49° E). Genomic DNA was isolated using modified CTAB protocol (Lodhi et al., 1994) and subsequently used for library construction including PacBio SMRTbell and Oxford Nanopore Technologies (ONT) ultra‐sequencing. For Hi‐C sequencing, the library construction was prepared using the NEBNext Ultra II kit, followed by sequencing on the Illumina NovaSeq platform (with PE150) (Rao et al., 2014). In addition, the apricot fruits were collected at five developmental stages (30, 45, 60, 75, 80 DPA). Fresh apricot flesh was sampled separately, cut into small pieces, immediately frozen in liquid nitrogen, and stored at −80°C until further analysis. Three biological replicates, each consisting of six fruits, were prepared for each sample to perform DNA methylation, metabolome, and transcriptome profiling.

### Genome assembly and gap filling

The clean Illumina data were used for k‐mer analysis to survey genome size and heterozygosity rate (Kajitani et al., 2014). The initial assembly was generated from PacBio HiFi reads using Hifiasm v0.16.1 with default parameters (Cheng et al., 2021). Subsequently, the high continuity of Nanopore ONT reads was combined with PacBio HiFi CCS reads for merged assembly using Hifiasm v0.16.1 and Verkko v1.4 with default parameters (Cheng et al., 2021; Rautiainen et al., 2023). The Hifiasm v0.16.1 assembly was selected as the better genome assembly and subjected to BLAST analysis against the NR database to remove plastid and contaminant sequences. The assembled contigs were anchored with Hi‐C reads by clustering the contig sequences into chromosome groups using a bottom‐up hierarchical clustering algorithm implemented in LACHESIS (Burton et al., 2013). The parameters were set as follows: minimum cluster restriction sites = 100, maximum link density = 2.5, and non‐informative ratio = 1.4 for clustering, and minimum number of restriction sites = 60 for both trunk and shredded contigs during ordering.

Gap filling was performed using ONT ultra‐long and PacBio HiFi data aligned with minimap2 v2.2.6 (‐a ‐‐MD ‐t 32 ‐‐secondary = no ‐k 19 ‐w 19) (Li, 2018). The alignments spanning both gap‐flanking regions were retained, and the longest alignment was used to fill the gap region in the genome. HiFi reads were then aligned to the gap‐filled assembly for validation. The assembly was further refined and polished with NextPolish2 v1.4.1 (hifi_options = ‐min_read_len 1 k ‐max_read_len 150k, hifi_minimap2_options = ‐x asm20) and manually inspected in Integrative Genomics Viewer (IGV) v2.13 (Hu et al., 2024; Thorvaldsdóttir et al., 2013). Meanwhile, to assess assembly completeness and coverage depth, Illumina paired‐end reads, HiFi reads, and ONT ultra‐long reads were independently mapped to the T2T genome assembly utilizing Minimap2 v2.2.6 (Li, 2018). BUSCO v4.0.5 and Merqury v1.3 were used to assess assembly completeness and Quality Value (QV), respectively (Rhie et al., 2020; Simão et al., 2015). BCFtools v1.8.0 and HISAT2 v2.1.0 were employed to estimate base accuracy and genes coverage, and BWA v0.7.8 was used for mapping Illumina paired‐end reads to genome assembly (Li & Durbin, 2010; Kim et al., 2015; Li, 2013).

### Telomere identification and centromere localization

Telomeres were identified through screening of telomeric repeats (AAACCCT) within a 2 kb flanking region adjacent to chromosomal termini. Telomere repeat units were analyzed using TIDK v0.2.0 (https://github.com/tolkit/telomeric‐identifier). A whole‐genome search was then performed with the following parameters: tidk search ‐f hap.fa ‐s TTTAGGG ‐o tidk_search ‐‐dir telomere_find. Centromeric regions were localized through genome‐wide identification of tandem repeat sequences and their monomeric units, utilizing the tandem repeats finder (TRF) (Benson, 1999). To ensure specificity for centromere‐associated histone binding, tandem repeat monomers were selectively retained based on a stringent size criterion (100–200 bp), corresponding to the typical length of functional centromeric repeats. To eliminate sequence redundancy and enhance centromere localization accuracy, monomeric units were clustered using CD‐HIT v4.09 with a sequence identity threshold of 0.9 (−c 0.9) and a word length of 5 (−n 5) (Fu et al., 2012). Genomic regions exhibiting continuous high‐frequency repeat patterns were computationally defined as putative centromeric sequences, with the predominant monomeric unit within each locus designated as the canonical centromeric repeat monomer based on read depth thresholding (≥50× coverage). Read coverage analysis in centromeric regions was performed using SAMtools.

### Whole‐genome bisulfite sequencing and analysis

Bisulfite sequencing was performed on the Illumina HiSeq 2500 platform, and raw reads were filtered using Trimmomatic v0.36. Clean reads were aligned to our assembled apricot reference genome utilizing Bismark v0.23.0 (Krueger & Andrews, 2011), and only uniquely aligned reads were retained for downstream analyses. Conversion rates were calculated based on the unmethylated lambda bacteriophage. Methylation sites were identified using the following thresholds: sequencing depth ≥4 and Q‐value ≤0.05. The degree of methylation (mC) and unmethylated cytosine (umC) was calculated using following equation: ML = mC/(mC + umC), respectively (Zhang et al., 2013). Identification of DMRs was conducted using a similar method as in reference (Akalin et al., 2012; Park & Wu, 2016; Wu et al., 2015). Cytosines with a sequencing depth of at least four in all libraries were retained for analysis. DMRs were identified using a 200‐bp sliding window with a 50‐bp step size via the DSS v2.12.0 package (https://github.com/yingjchen/DSS‐v2.0/). Regions exhibiting a methylation difference greater than 0.1 between control and treatment groups were classified as hyper‐ or hypomethylated DMRs. Methylation patterns within 2 kb upstream and downstream of genes and TEs were calculated and visualized using ViewBS v0.1.10 (Huang et al., 2018).

### Yeast one‐hybrid assay

The *PaLAR* promoter fragment was cloned and inserted into the pGADT7 vector using *P. armeniaca* genomic DNA as template. Meanwhile, the full‐length coding sequences (CDS) of *PaTT2* were cloned and inserted into pGBKT7 vector using *P. armeniaca* cDNA as template. Subsequently, according to previous methods, the pGADT7‐His2::*proPaLAR* fused vector, respectively together with pGBKT7::*PaTT2* were co‐transformed into yeast Y187 competent cells and cultured at 30°C for 3 days (Wang et al., 2024). Then, the positive clones were screened using defective culture medium (SD/‐Trp/‐Leu/‐His) supplemented with 80 mM 3‐amino‐1, 2, 4‐triazole (3‐AT). The yeast cells co‐transformed pGBKT7::*lam* and pGADT7‐His2 vectors were a negative control. The yeast cells co‐transformed pGBKT7::*p53* and pGADT7‐His2 vectors were a positive control.

### Dual‐luciferase reporter assays

Dual‐luciferase assays were conducted in tobacco (*Nicotiana benthamiana*) leaves to test the transactivation activities of the candidate hub TFs on the target promoters, as reported previously (Jiang et al., 2017). The dual‐luciferase reporter assay experiment was carried out according to previous work (Xu et al., 2025). The full‐length CDS of *PaTT2* was respectively inserted into the pGreenII 62‐SK vector. The promoter fragment of *PaLAR* was inserted into the pGreenII 0800‐LUC vector as reporters. Then, the pGreenII 0800‐LUC::pro*PaLAR* recombinant plasmid, together with pGreenII 62‐SK::*PaTT2*, was transiently co‐transformed into the cells of *N. benthamiana* leaves. After 72 h, the living imaging analysis system (Plant View100; Berthold) and dual‐luciferase reporter assay system (Promega) were used to assess promoter activity. Tissues surrounding the injection sites were collected to measure relative LUC/REN activities using a dual‐luciferase detection kit (Promega, Madison, WI, USA).

### Other methods

Details of the methods for repeat identification and gene prediction, phylogenetic construction and divergence time estimation, genomic synteny and whole‐genome duplication (WGD), transcriptome sequencing and data analysis, widely targeted metabolome analysis, qRT‐PCR and statistics analysis are available in the supplementary methods (Appendix S1).

## AUTHOR CONTRIBUTIONS

YJ conceived and designed the research. XQ and YJ wrote and revised the manuscript. XQ, TZ, and MW performed the experiments. XQ, TZ, and TR conducted data analysis. XQ, YJ, GB, YZ, ZC, and XD undertook field research and sampling. ZL and MY provided valuable advice. All authors read and approved the final manuscript.

## CONSENT

The authors have nothing to report.

## CONFLICT OF INTEREST

The authors declare no conflicts of interest.

## Supporting information


**Appendix S1:** Supplementary methods.

**Table S1.** Data statistics of whole‐genome sequencing for *P. armeniaca* Fyx.
**Table S2.** Statistics for the *P. armeniaca* Fyx assembly.
**Table S3.** Statistics for the eight pseudochromosomes.
**Table S4.** Statistics for the *P. armeniaca* Fyx finaly assembly.
**Table S5.** Quality assessment of a gapless *P. armeniaca* Fyx genome assembly after polishing.
**Table S6.** Completeness of the T2T genome assembly measured by Benchmarking Universal Single‐Copy Orthologs (BUSCO).
**Table S7.** Long‐terminal‐repeat assembly index (LAI) analysis and Comparison of genomic features of apricot assemblies.
**Table S8.** Statistics of repetitive sequence classification from the *P. armeniaca* Fyx genome.
**Table S9.** Statistics of gene functional annotation in the *P. armeniaca* Fyx genome.
**Table S10.** Completeness of the assembly and the annotated genes measured by Benchmarking Universal Single‐Copy Orthologs (BUSCO).
**Table S11.** Statistics of TF in the *P. armeniaca* Fyx genome.
**Table S12.** Statistics of ncRNA in the *P. armeniaca* Fyx genome.
**Table S13.** Statistics of the telomere positions at both termini of chromosomes within *P. armeniaca* Fyx genome.
**Table S14.** Statistics of locations and lengths of centromeric regions across all 8 chromosomes.
**Table S15.** The list of genes predicted on centromeres.
**Table S16.** GO enrichment analysis of genes on centromeres.
**Table S17.** GO enrichment analysis of the expanded gene families of *P. armeniaca* Fyx genome.
**Table S18.** KEGG enrichment analysis of the expanded gene families of *P. armeniaca* Fyx genome.
**Table S19.** GO enrichment analysis of the contracted gene families of *P. armeniaca* Fyx genome.
**Table S20.** KEGG enrichment analysis of the contracted gene families of *P. armeniaca* Fyx genome.
**Table S21.** Structural‐variant detection between *P. armeniaca* Fyx and five Prunus species.
**Table S22.** Identification of metabolites and annotations in Metabolome.
**Table S23.** Classification of identified metabolites across five fruit developmental stages.
**Table S24.** Data statistics of RNA sequencing for *P. armeniaca* Fyx at five developmental stages.
**Table S25.** Clustering of differentially accumulated metabolites abundance based on mfuzz.
**Table S26.** Clustering of differentially expressed genes abundance based on mfuzz.
**Table S27.** Representative DAMs involved in fruit quality.
**Table S28.** Differentially expressed structure genes involved in sugar and acid biosynthesis.
**Table S29.** Data statistics of WGBS sequencing for *P. armeniaca* Fyx at three developmental stages.
**Table S30.** Different DNA methylation types in *P. armeniaca* Fyx at three developmental stages.
**Table S31.** The expression of representative genes encoding DNA methyltransferases and demethylases, and key genes involved in the RdDM pathway of three fruit developmental stages.
**Table S32.** Differentially accumulated metabolites involved in flavonoid biosynthesis.
**Table S33.** Differentially expressed structure genes involved in flavonoid biosynthesis.
**Table S34.** Characteristics of CHH‐type differentially methylated regions (DMRs) in the promoter regions of *PaLAR, PaDFR* and *PaTT2*.
**Table S35.** KEGG enrichment analysis of the modules MEblue.
**Table 36.** Primers used in this study.

**Figure S1.** Genome survey of *P*. *armeniaca* Fyx.
**Figure S2.** Genome‐wide high‐throughput chromosome conformation capture (Hi‐C) interaction.
**Figure S3.** Mapping locations of reads for gap filling of *P*. *armeniaca* Fyx.
**Figure S4.** Summary of sequencing read characteristics.
**Figures S5–S8.** Visualization of the predicted centromeric region on Chr01, Chr02, Chr03, Chr04, Chr05, Chr07 and Chr08, respectively. The triangular heatmap depicts intra‐haplotype sequence identity, color‐coded by percentage similarity.
**Figure S9.** Functional enrichment analyses of significantly changed gene families and distribution of gene duplication modes in *P*. *armeniaca* Fyx and related species.
**Figure S10.** Distribution of different classes of metabolites identified in *P. armeniaca* Fyx.
**Figure S11.** Volcano plots of DEGs in T2‐T5 vs T1. Each point represents an mRNA, colored by differential expression significance (blue: low, red: high).
**Figure S12.** Orthogonal partial least squares discriminant analysis (OPLS‐DA) of DAMs and volcano plots of DAMs for T2 vs T1, T3 vs T1, T4 vs T1, and T5 vs T1.
**Figure S13.** Pearson correlation coefficient among three biological replicates of T1, T3, and T5, respectively.
**Figure S14.** Weighted gene co‐expression network analysis (WGCNA) of RNA‐seq data.
**Figure S15.** Characterization of hyper‐ and hypo‐DMRs in the MEblue module.
**Figure S16.** Genome browser view of DNA methylation and expression levels of *PaTT2*.

## Data Availability

Whole‐genome sequencing data have been deposited in the NCBI Bioproject database under accession number PRJNA1311747. Raw RNA‐seq and WGBS data are available under accession numbers PRJNA1304197 and PRJNA1306689, respectively. Genome assembly, annotation files, and centromere sequences are available in Zenodo (https://doi.org/10.5281/zenodo.17905465). All other data supporting the findings of this study are provided within the manuscript and its Supplementary Information.
