# Supplementary material for: The telomere‐to‐telomere gap‐free reference genome and DNA methylome unveil epigenetic regulation of proanthocyanidin biosynthesis during apricot fruit development
Source: Plant J. 2026 Sep 22;127(6):e71138. doi: 10.1111/tpj.71138 (PMC13595490; doi:10.1111/tpj.71138)
Supplement: Supplementary file 1 [file TPJ-127-0-s001.docx]

**Supplementary Material**

**Supporting Figures (16 Figures, Figures S1-S16)**

**Supporting Tables (36 Tables, Tables S1-S36)**

**Supporting Figures**

**Figure S1** Genome survey of *P*. *armeniaca* Fyx.

**Figure S2** Genome-wide high-throughput chromosome conformation capture (Hi-C) interaction.

**Figure S3** Mapping locations of reads for gap filling of *P*. *armeniaca* Fyx.

**Figure S4** Summary of sequencing read characteristics. (A) Length distribution of ONT ultra-long reads. (B) Length distribution of PacBio HiFi reads. (C) Sequencing depth statistics for three types of reads.

**Figure S5-Figure S8** Visualization of the predicted centromeric region on Chr01, Chr02, Chr03, Chr04, Chr05, Chr07 and Chr08, respectively. The triangular heatmap depicts intra-haplotype sequence identity, color-coded by percentage similarity.

**Figure S9** Functional enrichment analyses of significantly changed gene families and distribution of gene duplication modes in *P*. *armeniaca* Fyx and related species. (A) The enrichment of significantly expanded gene families. (B) The enrichment of significantly contracted gene families. (C) Distribution of duplication modes *P. armeniaca* Fyx and related species. Duplicated genes were classified into whole-genome duplication (WGD)/segmental duplication (SD), dispersed duplication (DD), tandem duplication (TD), and proximal duplication (PD) categories. The percentage of each category represents its contribution to the duplicated gene pool.

**Figure S10** Distribution of different classes of metabolites identified in *P. armeniaca* Fyx.

**Figure S11** Volcano plots of DEGs in T2-T5 vs T1. Each point represents an mRNA, colored by differential expression significance (blue: low, red: high). Horizontal and vertical reference lines -Log10pvalue = 1.3 and |log2FC| = 1, respectively.

**Figure S12** Orthogonal partial least squares discriminant analysis (OPLS-DA) of DAMs and volcano plots of DAMs for T2 vs T1, T3 vs T1, T4 vs T1, and T5 vs T1. Q² indicates model predictive ability, while R²X and R²Y values closer to 1 indicate better model fit. Each point represents a metabolite, with color reflecting the significance of differential accumulation, from blue (less significant) to red (more significant).

**Figure S13** Pearson correlation coefficient among three biological replicates of T1, T3, and T5, respectively.

**Figure S14** Weighted gene co-expression network analysis (WGCNA) of RNA-seq data. (A) Identification of the soft threshold for WGCNA. (B) Dendrogram of module clustering. (C) Heatmap illustrating the correlation between modules and traits.

**Figure S15** Characterization of hyper- and hypo-DMRs in the MEblue module. (A) Composition of DMRs identified in T1 vs T3 and T1 vs T5 comparisons. (B) Distribution of hyper- and hypo-DMRs around genes and transposable elements (TEs). DMRs were identified in T3 and T5 relative to T1 and used in this analysis. The y-axis represents the percentage of DMR number.

**Figure S16** Genome browser view of DNA methylation and expression levels of *PaTT2*. The DNA methylation levels are represented by the height of the vertical bars in each track (CG: red; CHG: yellow; CHH: purple). Gray shaded areas indicate the locations of DMRs.

**Supporting Tables**

**Table S1** Data statistics of whole-genome sequencing for *P*. *armeniaca* Fyx.

**Table S2** Statistics for the *P*. *armeniaca* Fyx assembly.

**Table S3** Statistics for the eight pseudochromosomes.

**Table S4** Statistics for the *P*. *armeniaca* Fyx finaly assembly.

**Table S5** Quality assessment of a gapless *P*. *armeniaca* Fyx genome assembly after polishing.

**Table S6** Completeness of the T2T genome assembly measured by Benchmarking Universal Single-Copy Orthologs (BUSCO).

**Table S7** Long-terminal-repeat assembly index (LAI) analysis and comparison of genomic features of apricot assemblies.

**Table S8** Statistics of repetitive sequence classification from the *P*. *armeniaca* Fyx genome.

**Table S9** Statistics of gene functional annotation in the *P*. *armeniaca* Fyx genome.

**Table S10** Completeness of the assembly and the annotated genes measured by Benchmarking Universal Single-Copy Orthologs (BUSCO).

**Table S11** Statistics of TF in the *P. armeniaca* Fyx genome.

**Table S12** Statistics of ncRNA in the *P*. *armeniaca* Fyx genome.

**Table S13** Statistics of the telomere positions at both ends of chromosomes within *P*. *armeniaca* Fyx genome.

**Table S14** Statistics of locations and lengths of centromeric regions across all 8 chromosomes.

**Table S15** The list of genes predicted on centromeres.

**Table S16** GO enrichment analysis of genes on centromeres.

**Table S17** GO enrichment analysis of the expanded gene families of *P*. *armeniaca* Fyx genome.

**Table S18** KEGG enrichment analysis of the expanded gene families of *P*. *armeniaca* Fyx genome.

**Table S19** GO enrichment analysis of the contracted gene families of *P*. *armeniaca* Fyx genome.

**Table S20** KEGG enrichment analysis of the contracted gene families of *P*. *armeniaca* Fyx genome.

**Table S21** Structural-variant detection between *P*. *armeniaca* Fyx and five *Prunus* species.

**Table S22** Identification of metabolites and annotations in Metabolome.

**Table S23** Classification of identified metabolites across five fruit developmental stages.

**Table S24** Data statistics of RNA sequencing for *P. armeniaca* Fyx at five developmental stages.

**Table S25** Clustering of differentially accumulated metabolites abundance based on mfuzz.

**Table S26** Clustering of differentially expressed genes abundance based on mfuzz.

**Table S27** Representative DAMs involved in fruit quality.

**Table S28** Differentially expressed structure genes involved in sugar and acid biosynthesis.

**Table S29** Data statistics of WGBS sequencing for *P. armeniaca* Fyx at three developmental stages.

**Table S30** Different DNA methylation types in *P. armeniaca* Fyx at three developmental stages.

**Table S31** The expression of representative genes encoding DNA methyltransferases and demethylases, and key genes involved in the RdDM pathway of three fruit developmental stages.

**Table S32** Differentially expressed structure genes involved in flavonoid biosynthesis.

**Table S33** Differentially accumulated metabolites involved in flavonoid biosynthesis.

**Table S34** Characteristics of CHH-type differentially methylated regions (DMRs) in the promoter regions of *PaLAR*, *PaDFR* and *PaTT2*.

**Table S35** KEGG enrichment analysis of the modules MEblue.

**Table S36** Primers used in this study.

**Figure S1**

**Figure S2**

**Figure S3**

**Figure S4**

**Figure S5**

**Figure S6**

**Figure S7**

**Figure S8**

**Figure S9**

**Figure S10**

**Figure S11**

**Figure S12**

**Figure S13**

**Figure S14**

**Figure S15**

**Figure S16**
