# Supplementary material for: The telomere‐to‐telomere gap‐free reference genome and DNA methylome unveil epigenetic regulation of proanthocyanidin biosynthesis during apricot fruit development
Source: Plant J. 2026 Sep 22;127(6):e71138. doi: 10.1111/tpj.71138 (PMC13595490; doi:10.1111/tpj.71138)
Supplement: Supplementary file 2 [file TPJ-127-0-s002.docx]

**Supplementary Methods**

**Repeat identification and gene prediction**

Tandem repeat annotation was conducted using GMATA v2.2 and Tandem Repeats Finder v4.07b (TRF) (Benson 1999; Wang and Wang 2016). GMATA was applied to identify simple repeat sequences with default parameters, while TRF was used to detect all tandem repeat elements (Liu et al. 2020). Transposable elements (TEs) were comprehensively annotated through an integrated approach combining ab initio prediction and homology-based methods. Specifically, MITE-Hunter was utilized to classify and mask TEs based on known repeat libraries (Tarailo-Graovac and Chen 2009). To enhance annotation accuracy, a custom TE library was constructed by integrating consensus sequences generated by TEclass with curated repeat databases from RepeatMasker, enabling the detection of both conserved and lineage-specific TEs.

Non-coding RNAs (ncRNAs) screening was performed by querying the Rfam database (https://pfam.xfam.org/) using Infernal cmscan v1.1.4 with stringent parameters (--cut_ga --rfam --nohmmonly --fmt 2) to ensure homology detection based on covariance model (Nawrocki and Eddy 2013). tRNA annotation was conducted using tRNAscan-SE v2.0 with eukaryotic-specific settings (Lowe and Eddy, 1997). For miRNA, snRNA, snoRNA, and rRNA detection, an integrated approach was employed: Infernal cmscan identified structural motifs against Rfam models, and RNAmmer v1.2 predicted ribosomal RNAs using kingdom-specific HMM profiles (Lagesen et al. 2007). Gene annotation was conducted through an integrated pipeline combining three complementary approaches. *De novo prediction* utilizing hidden Markov models to identify putative gene structures based on intrinsic genomic features. Gene prediction was performed using AUGUSTUS v3.3.1 under ab initio mode, with a species-specific training set derived from transcriptome evidence (Stanke et al. 2008). Homology-based annotation via GeMoMa v1.6.1, which aligned conserved peptide sequences from reference species to infer exon-intron boundaries and splice variants, with parameter optimization for eukaryotic genomes (Keilwagen et al. 2019). RNA-seq evidence integration, where the filtered mRNA-seq reads were mapped to the genome using STAR v2.7.3a to validate transcriptional isoforms and refine gene models (Dobin et al. 2013). Open reading frames (ORFs) were subsequently predicted through PASA v2.3.3. The consensus gene set was generated by EVidenceModeler v1.1.1, which integrated ab initio predictions, protein homology alignments, and transcriptome evidence through a weighted evidence-based framework (Haas et al. 2008). Gene function annotation, conserved motif identification, and domain prediction were performed through integrated methods. For functional annotation, SwissProt was employed for high-confidence functional assignments, Non-redundant (NR) database was used for broad-spectrum homology searches, and KEGG for pathway enrichment of metabolic and signaling pathways, KOG/COG databases for clustering orthologous groups, and Gene Ontology terms for hierarchical categorization of molecular functions, biological processes, and cellular components. Moreover, genomic synteny and compositional features were visualized using Circos v0.69 (Krzywinski et al. 2009).

**Phylogenetic construction and divergence time estimation**

To elucidate genomic evolution, we compiled a comparative dataset comprising the protein and coding sequences from *P. armeniaca* Fyx and 14 relative or phylogenetically representative species spanning Rosaceae (*Malus domestica*, *Rosa chinensis*, *R*. *rugosa*), *Prunus sensu lato* (*P*. *avium*, *P*. *armeniaca*, *P*. *persica*, *P*. *mongolica*, *P*. *salicina*, *P*. *brigantina*, *P*. *dulcis*, *P*. *campanulata*), representative eudicots (*Vitis vinifera*), monocots (*Oryza sativa*), and the outgroup *Morus notabilis* (Moraceae). Protein orthogroups were inferred using OrthoFinder v2.3.3 with default parameters (Emms and Kelly 2019). Protein sequences derived from single-copy gene families across all 15 species were aligned using MAFFT v7.313. Using the aligned data, maximum likelihood phylogenetic trees were constructed with RAxML v8.2, under 1000 bootstrap replicates (Stamatakis 2014). The tree file was visualized using iTOL (https://itol.embl.de/). Subsequently, divergence times and branch-specific substitution rates were estimated employing the MCMCTree program, utilizing the reconstructed phylogeny as the reference topology. Three fossil-based divergence time constraints from the TimeTree database (http://www.timetree.org/) were used to calibrate the molecular clock analysis. These calibrations consisted of: (i) the monocot-dicot divergence (*V*. *vinifera*–*O*. *sativa*) constrained between 142–163 Mya, (ii) the Moraceae-Vitaceae split (*M*. *notabilis*–*V*. *vinifera*) bounded at 109.8–124.4 Mya, and (iii) the Rosaceae-Moraceae divergence dated to 76.0–95.5 Mya. To elucidate the evolutionary dynamics of gene families, the analysis of gene family expansion and contraction was conducted. The orthogroup matrix generated by OrthoFinder served as input data for the CAFÉ program to quantify lineage-specific changes in gene copy numbers (De Bie et al. 2006). Lineage-specific gene family size changes were inferred based on λ parameters derived from CAFÉ’s stochastic birth-death framework. Significant gene family changes (expansion/contraction) were designated based on a *P*-value threshold of 0.05, followed by functional profiling of these gene sets via GO and KEGG enrichments.

**Genomic synteny and whole-genome duplication (WGD)**

Genome-wide syntenic blocks were delineated with JCVI v1.1.7, implementing the Python adaptation of MCScanX, to investigate collinear relationships across *P. armeniaca* Fyx, *V. vinifera*, *M. domestica*, and *P. armeniaca*. Syntenic regions were delineated subject to minspan = 30 (Wang et al. 2012). To investigate WGD events in P. armeniaca Fyx, synonymous substitution rates (Ks) were calculated using wgd software v1.1.3 (https://github.com/arzwa/wgd/), and fitted Ks distributions for paralogous gene pairs were delineated with R v4.3.1. Structural variants (SVs) between *P. armeniaca* Fyx and other five related *Prunus* genomes (*P. armeniaca*, *P. zhengheensis*, *P. mume*, *P. dulcis*, and *P. salicina* Fengtangli) were identified using MUMmer v3.23 and validated through SyRI (Goel et al. 2019).

**Transcriptome sequencing and data analysis**

Total RNA was extracted from apricot flesh samples collected at five developmental stages (30, 45, 60, 75, 80 DPA). RNA library construction and sequencing were conducted by Wuhan Metware Biotechnology Co., Ltd (Wuhan, China). Briefly, RNA-seq libraries were sequenced using the Illumina HiSeq-PE150 platform. Raw reads were subjected to quality control and base correction using FastQC v0.11.9 (Andrews et al. 2010) and then the final high quality cleaned reads were aligned to our assembled apricot genome using HISAT2 v2.0.5 (Kim et al. 2015) with default parameters. Gene expression was quantified for each sample using HTSeq v0.7.2 (Anders et al. 2010), and FPKM values were calculated based on gene length. PCA and correlation coefficients between samples were performed to compare the expressed gene profiles among developmental stages using the PRCOMP function in R package ade4 v1.7-23 and pheatmap v1.0.13. DESeq2 v1.18.0 was used to identify the DEGs according to a fold change ≥ 2 and pvalues < 0.05 (Love et al. 2014). The mfuzz function in R package ClusterGVis v0.14 was used to exhibit clustering and visualization of of all DEGs expression at different periods (Zhang et al. 2022). GO and KEGG enrichment analyses were performed using the R package clusterProfiler v4.16.0 and ggplot2 v3.5.2 (Wickham et al. 2016; Wu et al. 2021). Gene set enrichment analysis (GSEA) was conducted on the DEGs to assess the enrichment of KEGG pathways, using ClusterGVis (https://github.com/junjunlab/ClusterGVis) for computation and visualization. Additionally, ITAK (http://itak.feilab.net/cgi-bin/itak/index.cgi) was employed to predict TFs.

The FPKM values of all DEGs together with the quantified levels of key metabolites (e.g., sugars, organic acids, flavonoids) across the fruit developmental stages were utilized to construct a gene co-expression network using WGCNA v1.73 (Langfelder et al. 2008). The soft-thresholding power was set to 8, with a scale-free model fitting index R² > 0.8. Meanwhile, we also select the large minimum module size (30), the cutHeight (0.25) and eamedium sensitivity (deepSplit=2) to cluster splitting models. The co-expression module (MEblue), along with hub metabolites, candidate TFs, and structural genes determined based on Pearson’s correlation coefficients, was integrated into a final network and visualized using Cytoscape v3.8.2 (Otasek et al. 2019).

**Widely targeted metabolome analysis**

Apricot pulp samples were extracted and analyzed by Wuhan Metware Biotechnology Co., Ltd. (Wuhan, China) according to the standard protocols (Chen et al. 2013; Fraga et al. 2010). Metabolite profiling was performed using a UPLC-ESI–MS/MS system, and raw mass spectrometry data were processed in Analyst v1.6.3. Then, the metabolites were characterized based on secondary spectral information and annotated using a self-constructed Metware database. Differentially accumulated metabolites (DAMs) were identified using criteria of variable importance in projection (VIP) ≥ 1 and fold change ≥ 2 or ≤ 0.5. To generate non-redundant datasets, DAMs and DEGs identified in multiple pairwise comparisons were merged, and overlapping metabolites or genes were counted only once.

**Quantitative real-time PCR (qRT-PCR) analysis**

To validate gene expression, transcript levels of six anthocyanin biosynthesis genes in the MEblue module during apricot storage were quantified by qRT-PCR using the 2^−ΔΔ^CT method, according to previously established protocols (Zhang et al. 2019). Total RNA was extracted from apricot pulp using the RNAprep Pure Plant Kit (Tiangen Biotech, Beijing, China). qRT-PCR was carried out with TB Green Premix Ex Taq II (Takara, Japan) on a Bio-Rad IQ5 Real-Time System (Bio-Rad, Hercules, CA). Actin was used as the reference gene due to its stable expression across apricot ripening stages (Chen et al. 2023). The primers used for qRT-PCR are listed in Table S36. Differences between treatments and their relevant controls were evaluated using paired two-tailed Student’s t-tests in R, with *P* ≤ 0.05 considered statistically significant.

**Reference**

Anders, S., Pyl, P.T. & Huber, W. (2010) HTSeq: analysing high-throughput sequencing data with Python. *Bioinformatics*, 31, 166–169. https://doi.org/10.1093/bioinformatics/btu638

Andersson, R., Gebhard, C., Miguel-Escalada, I., Hoof, I., Bornholdt, J., Boyd, M., et al. (2010) FastQC: a quality control tool for high throughput sequence data. Version 0.11.2.

Benson, G. (1999) Tandem repeats finder: a program to analyze DNA sequences. *Nucleic Acids Research*, 27, 573–580. https://doi.org/10.1093/nar/27.2.573

Chen, W., Gong, L., Guo, Z., Wang, W., Zhang, H., Liu, X., et al. (2013) A novel integrated method for large-scale detection, identification, and quantification of widely targeted metabolites: application in the study of rice metabolomics. *Molecular Plant*, 6, 1769–1780. https://doi.org/10.1093/mp/sst080

Chen, Y., Li, W., Jia, K., Liao, K., Liu, L., Fan, G., et al. (2023) Metabolomic and transcriptomic analyses of flavonoid biosynthesis in apricot fruits. *Frontiers in Plant Science*, 14, 1210309. https://doi.org/10.3389/fpls.2023.1210309

De Bie, T., Cristianini, N., Demuth, J.P. & Hahn, M.W. (2006) CAFE: a computational tool for the study of gene family evolution. *Bioinformatics*, 22, 1269–1271. <https://doi.org/10.1093/bioinformatics/btl097>

Dobin, A., Davis, C.A., Schlesinger, F., Drenkow, J., Zaleski, C., Jha, S., et al. (2013) STAR: ultrafast universal RNA-seq aligner. *Bioinformatics*, 29, 15–21. https://doi.org/10.1093/bioinformatics/bts635

Emms D.M. & Kelly, S. (2019) OrthoFinder: phylogenetic orthology inference for comparative genomics. *Genome Biology*, 20, 238. https://doi.org/10.1186/s13059-019-1832-y

Fraga, C.G., Clowers, B.H., Moore, R.J. & Zink, E.M.. (2010) Signature-discovery approach for sample matching of a nerve-agent precursor using liquid chromatography–mass spectrometry, XCMS, and chemometrics. *Analytical Chemistry*, 82, 4165–4173. https://doi.org/10.1021/ac1003568

Goel, M., Sun, H., Jiao, W.B. & Schneeberger, K. (2019) SyRI: finding genomic rearrangements and local sequence differences from whole-genome assemblies. *Genome Biology*, 20, 277. https://doi.org/10.1186/s13059-019-1911-0

Haas, B.J., Salzberg, S.L., Zhu, W., Pertea, M., Allen, J.E., Orvis, J., et al. (2008) Automated eukaryotic gene structure annotation using evidence modeler and the program to assemble spliced alignments. *Genome Biology*, 9, R7. https://doi.org/10.1186/gb-2008-9-1-r7

Keilwagen J., Hartung F., & Grau J. (2019) GeMoMa: homology-based gene prediction utilizing intron position conservation and RNA-seq data. In: *Gene Prediction* (Kollmar, M., ed.), Methods in Molecular Biology, vol. 1962. New York: Humana.

Kim, D., Langmead, B. & Salzberg, S.L. (2015) HISAT: a fast spliced aligner with low memory requirements. *Nature Methods*, 12, 357–360. https://doi.org/10.1038/nmeth.3317

Krzywinski, M., Schein, J., Birol, I., Connors, J., Gascoyne, R., Horsman, D. et al. (2009) Circos: an information aesthetic for comparative genomics. *Genome Research*, 19, 1639–1645. https://doi.org/10.1101/gr.092759.109

Lagesen, K., Hallin, P., Rødland, E.A., Staerfeldt, H.H., Rognes, T., & Ussery, D.W. (2007) RNAmmer: consistent and rapid annotation of ribosomal RNA genes. *Nucleic Acids Research*, 35, 3100–3108. https://doi.org/10.1093/nar/gkm160

Langfelder, P. & Horvath, S. (2008) WGCNA: an R package for weighted correlation network analysis. *BMC Bioinformatics*, 9, 559. https://doi.org/10.1186/1471-2105-9-559

Liu, C., Feng, C., Peng, W., Hao, J., Wang, J., Pan, J., et al. (2020) Chromosome-level draft genome of a diploid plum (*Prunus salicina*). GigaScience 9, giaa130. https://doi.org/10.1093/gigascience/giaa130

Love, M.I., Huber, W. & Anders, S. (2014) Moderated estimation of fold change and dispersion for RNA-seq data with DESeq2. *Genome Biology*, 15, 550. <https://doi.org/10.1186/s13059-014-0550-8>

Nawrocki, E.P. & Eddy, S.R. (2013) Infernal 1.1: 100-fold faster RNA homology searches. *Bioinformatics*, 29, 2933–2935. https://doi.org/10.1093/bioinformatics/btt509

Otasek, D., Morris, J.H., Bouças, J., Pico, A.R., & Demchak, B. (2019) Cytoscape Automation: empowering workflow-based network analysis. *Genome Biology*, 20, 185. https://doi.org/10.1186/s13059-019-1758-4

Stamatakis, A. (2014) RAxML version 8: a tool for phylogenetic analysis and post-analysis of large phylogenies. *Bioinformatics*, 30, 1312–1313. https://doi.org/10.1093/bioinformatics/btu033

Stanke, M., Diekhans, M., Baertsch, R., & Haussler, D. (2008) Using native and syntenically mapped cDNA alignments to improve de novo gene finding. *Bioinformatics*, 24, 637–644. https://doi.org/10.1093/bioinformatics/btn013

Tarailo-Graovac, M., & Chen, N. (2009) Using RepeatMasker to identify repetitive elements in genomic sequences. *Current Protocols in Bioinformatics*, Chapter 4, 4.10.1–4.10.14. https://doi.org/10.1002/0471250953.bi0410s25

Wang, X.W. & Wang, L. (2016) GMATA: an integrated software package for genome-scale SSR mining, marker development and viewing. *Frontiers in Plant Science*, 7, 1350. https://doi.org/10.3389/fpls.2016.01350

Wang, Y.P., Tang, H.B., DeBarry, J.D., Tan, X., Li, J.P., Wang, X.Y. et al. (2012) MCScanX: a toolkit for detection and evolutionary analysis of gene synteny and collinearity. *Nucleic Acids Research*, 40, e49. https://doi.org/10.1093/nar/gkr1293

Wickham H., Chang W. & Wickham M.H. (2016) Package ‘ggplot2’: create elegant data visualisations using the grammar of graphics. Version 2(1), 1–189.

Wu, T., Hu, E., Xu, S., Chen, M., Guo, P., Dai, Z., et al. (2021) clusterProfiler 4.0: a universal enrichment tool for interpreting omics data. *Innovation*, 2, 100141. https://doi.org/10.1016/j.xinn.2021.100141

Zhang, Q., Qi, Y., Li, R., Yang, Y., Yan, D., Liu, X., et al. (2019) Postharvest applications of n-butanol increase greasiness in apple skins by altering wax composition via effects on gene expression. *Postharvest Biology and Technology*, 155, 111–119. https://doi.org/10.1016/j.postharvbio.2019.05.017
